# Impact of caregiver incentives on child health: Evidence from an experiment with Anganwadi workers in India

**DOI:** 10.1016/j.jhealeco.2017.07.005

**Published:** 2017-09

**Authors:** Prakarsh Singh, William A. Masters

**Affiliations:** a Amherst College, United States; b Tufts University, United States

**Keywords:** Performance pay, Malnutrition, Underweight, Child development

## Abstract

This paper tests the effectiveness of performance pay and bonuses among government childcare workers in India. In a controlled study of 160 ICDS centers serving over 4000 children, we randomly assign workers to either fixed bonuses or payments based on the nutritional status of children in their care, and also collect data from a control group receiving only standard salaries. In all three study arms mothers receive nutrition information. We find that performance pay reduces underweight prevalence by about 5 percentage points over 3 months, and height improves by about one centimeter. Impacts on weight continue when incentives are renewed and return to parallel trends thereafter. Fixed bonuses are less expensive but lead to smaller and less precisely estimated effects than performance pay, especially for children near malnutrition thresholds. Both treatments improve worker effort and communication with mothers, who in turn feed a more calorific diet to children at home.

Performance pay and financial incentives for health improvements are controversial. Well-targeted incentives may improve efficiency, but can also crowd out intrinsic motivation leading to worse results than unconditional payments that elicit voluntary efforts. Previous studies on incentives to improve health in developing countries have focused primarily on payments to individuals for their own health as in Powell-Jackson et al. (2015) or to caregivers to provide specific services as in De Walque et al. (2015). Here we provide incentives to health workers based on client outcomes, targeting weight-for-age malnutrition among children attending urban day care centers in Chandigarh, India. The trial builds on Singh, 2015, Singh, 2016, in collaboration with local health authorities to improve services in an urban area where about 40% of children are clinically underweight.

The economic value of preventing child malnutrition is the subject of a long literature in economics, from Strauss (1986) to Scholte et al. (2016), Berg et al. (2013) and many others. Following Behrman and Deolalikar (1987), improvements over time have been found to be driven primarily by technological and institutional changes that shift food demand and care practices. In India, one factor influencing child nutrition is performance of preschool daycare centers funded through the government’s Integrated Child Development Services (ICDS) program. This is the world's largest child development system, launched in 1975 to help prevent malnutrition, which ICDS managers typically measure in terms of low weight for age. There are now over 1.3 million ICDS centers across the country, each providing daycare for about 30 children from 6 months to 6 years of age through an Anganwadi worker who is paid a fixed salary of approximately Rs. 4000 or US$67 per month. ICDS sites are also used to provide supplemental nutrition via take-home rations for pregnant women, lactating mothers and any child below 6 years of age (ICDS, 2017). At our sites the older children above 3 years of age are expected to attend the center from 9:00 am to noon, and to receive a mid-day meal provided by the caregiver. Other services provided include immunization, health checkups and nutrition advice.

The quality of services actually delivered at ICDS centers is highly contentious. Program gaps exist in almost all areas of ICDS delivery, and even basic amenities are often lacking (Chudasama et al., 2014; Gupta et al., 2013). Workers have limited education, training, job security and infrastructure (Ramachandran et al., 2010; Sharma et al., 2014), and they report significant stress and dissatisfaction (Mohanan et al., 2012). If motivated to do so, caregivers can influence the prevalence of child underweight through two main channels: first through the direct provision of food and other services to the child at the center, and secondly through effective communication with mothers either when they drop off or pick up their child from the center or by making home visits. A World Bank report by Gragnolati et al. (2005) found both to be limited, with many missing meals and almost no effective communication between workers and mothers. A more recent household survey in 100 Indian districts indicates that although 96 percent of locations were served by a functioning ICDS center, only 50 percent of them actually provided food on the day of survey and just 19 percent of the mothers reported that the workers provides nutrition counselling (Hungama Report, 2011).

Previous studies of ICDS program impacts have used cross-sectional observations and found mixed results: Deolalikar (2005) found evidence of lower underweight prevalence among boys in locations with ICDS centers in the early 1990s, but Lokshin et al., (2015) find strong selection effects with ICDS centers more likely to be located in places that also benefit from other advantages. Focusing within Chandigarh, Thakur et al. (2011) found that program enrollment was not associated with lower prevalence of underweight children, but using national data and matching methods to address geographic and household-specific selection effects Kandpal (2011) and Jain (2015) found significant gains in child height, and Mittal and Meenakshi (2015) found reductions in the prevalence of underweight. Randomized trials at ICDS centers have focused on introducing additional services, such as increasing take-home rations (Avula et al., 2011).

In this study we evaluate the effectiveness of targeting workers’ efforts using existing resources, through performance pay or fixed bonuses of the order of 2–5 percent above the worker’s regular salary. Our bonus is based on the principal nutritional objective of ICDS services which is to reduce the fraction of children who are classified as underweight. Other data we collect allow us to look beyond average treatment effects on underweight status, examining the degree to which caregivers target efforts at children near classification thresholds and also capturing impacts on linear growth through changes in height. We consider the timing of change including whether gains persist after performance pay is discontinued, and we address the mechanisms by which changes are achieved, using unannounced visits to each center to observe workers’ level of effort plus detailed household and caregiver surveys at regular intervals to study behavioral responses.

A growing literature finds that small changes in compensation schemes and recognition for specific achievements can generate substantial improvements in performance, at a fraction of the cost of funding additional services or a “business as usual” budget increases (Ratto and Burgess, 2003, Glewwe and Muralidharan, 2015). A central challenge in these schemes is to align workers’ incentives with policymakers’ objectives. In our setting, each Anganwadi worker is individually responsible for services at her center, thereby avoiding the problems associated with free-ridership in groups identified by Burgess et al. (2017). We take advantage of this structure to make payments based on overall outcomes among all the children at each center, incentivizing each worker to allocate efforts among tasks in somewhat the same way as payments to teachers for education outcomes based on improved test scores (Behrman et al., 2012, Muralidharan and Sundararaman, 2011, Hasnain et al., 2014). Focusing on outcomes creates incentives for workers to employ contextual knowledge about the optimal allocation of available resources, as for example when performance incentives to primary school principals in China led them to make changes that reduced child anemia (Miller and Babiarz, 2014, Miller et al., 2015, Miller et al., 2012). In contrast to those outcome-based payments in education, performance pay in the health sector usually targets the provision of intermediate inputs such as clinic visits or other specific services (Banerjee et al., 2010, Basinga et al., 2011).

Health outcomes like child underweight depend not only on the caregiver’s actions, but also on external factors such as the child’s diet at home as well as their sanitation and disease environment (Hammer and Spears, 2016). Like Gertler and Vermeersch (2012), our study aims to test performance incentives with no change in those external factors, for a given level of resources available to caregivers and households. The only input we provide other than the incentive itself is a simple and inexpensive recipe book with nutrition information, which caregivers and mothers could use to improve care if they choose to do so. Singh (2015) found that this very low-cost information treatment complements caregiver efforts, by facilitating communication about how to prepare safe, nutrient-dense meals. We distribute the books to all mothers of children in the trial, and also inform ICDS workers about the book so they can refer to it and discuss specific recipes or the effects of disease and sanitation on child health, based on the caregiver’s localized knowledge of the family’s circumstances.

Designing incentives for health workers to use existing resources most effectively has long been and remains challenging across a wide range of settings (Hillman et al., 1998, Burgess and Metcalfe, 1999, Mannion and Davies, 2008). Explicit incentives can crowd out other motivations (Lazear, 2000, Benabou and Tirole, 2003, Benabou and Tirole, 2006, Ashraf et al., 2014), competition can discourage entry (Cason et al., 2010), and after the scheme is discontinued performance may be worse than before it was introduced (Camerer, 2010, Lester et al., 2010). Our test is designed to address these concerns, allowing comparison over time between performance payments, a fixed bonus treatment that is calibrated to have about the same expected value, and a control group in a different part of the city that receives no payments above their base salary. The comparison between treatment groups isolates the impact of making payments conditional on measured outcomes, as opposed to relying on workers’ intrinsic motivation to improve measured outcomes as in Bhushan et al. (2007). We also compare both payment arms to a pure status-quo block in which Anganwadi workers receive only their base salary, to test the effects of any kind of payment and targeting relative to city-wide trends, seasonality and price shocks.

An important feature of our design is to compare performance pay to fixed bonuses, testing the strength of gift exchange (Akerlof, 1982, Akerlof, 1984) as a kind of efficiency wage by which employers offer compensation above market wages to elicit voluntary efforts in return (Montinari et al., 2016). Baron (2013) uses the term “empathy” wages to distinguish the activation of social norms or moral obligations from other kinds of voluntary effort, as when efficiency wages are used to facilitate sorting, limit turnover and build reputation (Fehr et al., 1993; Fehr and Gächter, 2001). Several studies suggest that unconditional bonuses can outperform conditional incentives in improving worker effort.^1^ Our experiment compares them to each other, and to outcomes in a control group where workers are given no additional motivation to achieve improved outcomes. Eliciting greater voluntary efforts is especially important in settings such as the ICDS centers where public-sector workers operate under limited supervision, and must allocate effort among tasks whose costs and payoffs are heterogeneous and difficult to observe. These choices are spelled out in the model below.

## Conceptual framework

Each caregiver in our trial can be seen as allocating her efforts between multiple tasks serving multiple children. For simplicity, we focus here on her allocation of effort between two children, denoting net utility for caregiver *i* as $V_{i}\left(x_{i1},x_{i2}\right)$, where *x_ij_* is her marginal cost of effort for child *j*. The caregiver’s utility may be derived from her baseline intrinsic motivation to help each child, *γ_ij_*, net of a convex cost function that we posit to be $0.5\left(x_{ij}\right)$^2^ over each level of effort. Financial incentives are a baseline salary, plus either a fixed bonus *K_i_* or a performance payment *π_ij_* that is based on observed outcomes *f*(*x*_ij_) from the worker’s efforts for each child. With that notation, the three incentive schemes in our trial can be represented as follows:

### Status quo: wage employment

In our setting, all employees have secure employment so their efforts are motivated by intrinsic motivation to help each child, minus their cost of effort:

$$V_{i}\left(x_{1},x_{2}\right)=\gamma_{i1}.x_{1}+\gamma_{i2}.x_{2}-0.5{\left(x_{i1}\right)}^{2}-0.5{\left(x_{i2}\right)}^{2}$$

The caregiver’s baseline motivations, *γ_i_*_1_ and *γ_i_*_2_, could be different for the two children and costs *x_i_*_1_ and *x_i_*_2_ could also differ, for example based on the gender, caste or socioeconomic status of the child, as well as the caregiver’s circumstances. With this specification, the only driving force for workers to exert effort is their intrinsic motivation, so the optimal allocation of effort equates marginal cost of effort with marginal utility of helping for each child:

$$x_{ij}=\gamma_{ij}$$

### Performance pay: wage employment + πfor each grade improvement

Offering performance pay might displace intrinsic motivations by an amount μ*_ij_*, while adding a payment for each improvement actually observed. With risk neutrality, the utility of those payments depends on the conditional probability that improvement will occur, $Prob\left(Imp_{j}\right)$:

$$V_{i}\left(x_{1},x_{2}\right)=\left(\gamma_{i1}-\mu_{i1}\right)x_{1}+(\gamma_{i2}-\mu_{i2})x_{2}-0.5{\left(x_{1}\right)}^{2}-0.5\;{\left(x_{2}\right)}^{2}+\;\left(Prob\left(Imp_{1}\right)*\pi \right)+\left(Prob\left(Imp_{2}\right)*\pi \right)$$

In our setting, the probability of improvement would be a function of the worker’s effort and child characteristics, including distance from the child’s current status to the child-specific threshold over which improvements would be counted: $Prob\left(Imp_{j}\right)$ = *f*(*x*_ij_, $\bar{w_{j}}$-w_j_). Predicted effort in response to performance pay thus depends on the marginal payoff from increased effort, relative to intrinsic motivations:

*x*_1_ = *γ*_*i*1_ − μ_*i*1_ + *πf’*(*x*_1_); *x*_2_ = *γ*_*i*2_ − μ _*i*1_ + *πf’*(*x*_2_)

One testable prediction of this model is that caregivers offered these incentives will target additional efforts towards children with higher probability of improvement, for example due to initial measurements closer to the threshold. With limits on the total effort that can be exerted (for example, if *x*_1_ +*x*_2_ $\leq \bar{x}$), this could increase the dispersion of effort expended among children as in Neal and Schanzenbach (2010).

### Fixed bonus: wage employment + payment of *K(i)* unrelated to child *j*

When payments are not based on each child’s outcomes, they operate only by altering intrinsic motivations:

$$V_{i}\left(x_{1},x_{2}\right)=\left(\gamma_{i1}+K_{i1}\right)x_{1}+(\gamma_{i2}+K_{i2})x_{2}-0.5{\left(x_{1}\right)}^{2}-0.5{\left(x_{2}\right)}^{2}$$

Now the solution is a modification of our baseline case:

$$x_{1}=\gamma_{i1}+K_{i1};:x_{2}=\gamma_{i2}+K_{i1}$$

The baseline level of intrinsic motivation, and the added voluntary effort triggered by gift exchange, could be child-specific and depend on the child’s distance from their threshold, but we would expect a smaller threshold effect. The previous literature cited above suggests that voluntary efforts can be elicited through gift exchange while performance pay can crowd out intrinsic motivations, so the relative performance of these two schemes is an empirical question. The only unambiguous prediction is that performance pay would elicit more effort and hence more improvements than the fixed bonus among children who are closer to their nearest threshold at baseline.

## Treatments

The performance pay treatment we offer reflects the stated objective of the ICDS program, which is to reduce the number of children classified as severely or moderately malnourished in terms of weight for age. In the incentive treatment, workers were offered a bonus of Rs. 200 (about US$3) for each child whose classification improved, net of any children whose classification worsened, over each three-month round of the trial. The alternative fixed bonus was set at Rs. 200 over three months, reflecting the expected performance of one net improvement observed in the earlier Chandigarh and Kolkata performance pay schemes in Singh (2015) and Singh and Mitra (2017). Payoffs in the performance-pay treatment are truncated at zero, as no money would ever be taken from workers should the children in their center experience more declines than advances over the three-month period.

To ensure clarity regarding program objectives, each ICDS worker in the trial was provided a goal card with lists of her enrolled children, their present health indicators and target weights after three months. Each target was calculated on the basis of the World Health Organization (WHO) reference levels of weight for severely, moderately and not malnourished children of each age (in months) and sex. Target weights were the WHO thresholds for improvement (or worsening) from one category to the next.

None of the children in these ICDS centers was at risk of becoming overweight in terms of weight-for-age, but some were short enough that weight increases to achieve a normal weight-for-age could make them overweight by the WHO’s criterion of more than one standard deviation above normal weight-for-height at that age and sex. To avoid incentivizing excess weight gain in these cases, target weights were reduced to that threshold for those who were either moderately malnourished (17 children) or severely malnourished (7 children), so that achieving the target would not make the child overweight for their height.

Caregivers in the treatment arms with the fixed bonus of Rs. 200 were also provided with goal cards noting target weights for all children, and mothers in all treatment arms were provided recipe books to help them respond to ICDS caregivers’ efforts following Singh (2016). The weight and height measurements of children were conducted at each site independently of ICDS management, by a team of hired enumerators. For a random sample of children, weights were also cross-checked by hired supervisors. This arrangement ensures that the impact of treatments we provide would be adequately measured and feasible for the ICDS to scale up or test elsewhere, as the goal cards for each caregiver, recipe books for each mother, and incentive payments of Rs. 200 over three months are relatively low cost compared to workers’ monthly salary of approximately Rs. 4000. The low magnitude of incentives and independent measurement on varied occasions also help to reduce the chance of workers “gaming” the system, while repeated measurements allow us to check for parallel trends across treatment and control groups before incentives are introduced and after they are withdrawn.

## Experimental design and data collection

**Fig. 1:**
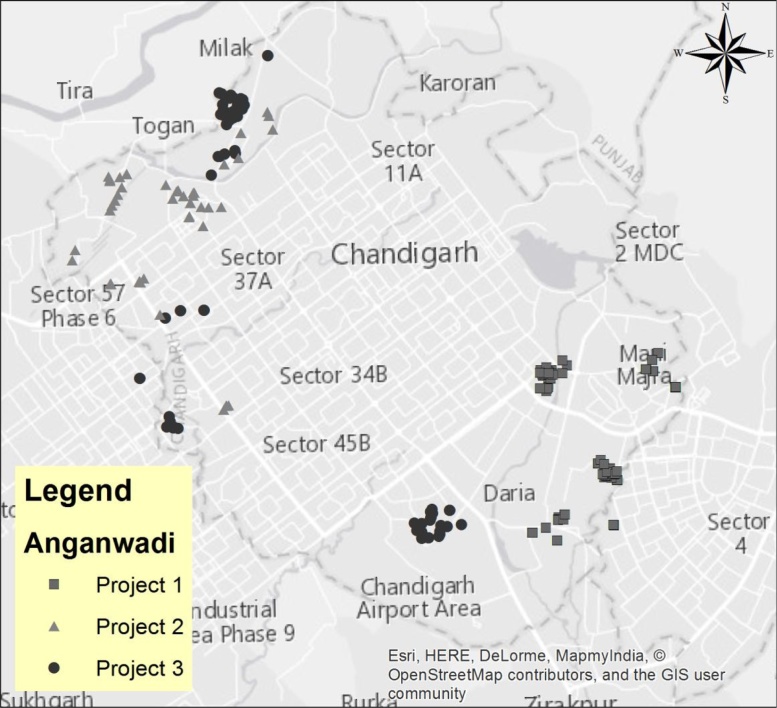
Participating Anganwadi centers in Chandigarh are located in three administratively and geographically distinct project blocks.

Our project was carried out in close collaboration with the Social Welfare Department of Chandigarh, a Union Territory in northern India. As shown in Fig. 1, we draw ICDS centers from geographically separate parts of the city administered by different block officers, to preclude spillovers between the treatment arms and the control group. Table 1 shows the timeline of the experiment, involving a sequence of month-long surveys to measure all children in each center at intervals of three months, in July and October 2014, and then in January, April and July 2015. In addition, there were unannounced supervisory visits to the centers between rounds to measure attendance of workers and monitor effort. During the first three-month period between two baseline surveys no treatments were provided, so as to test for pre-treatment differences in time trends among the sites. We also continued two rounds of observation after the treatment, to test for persistence of impacts and any possible negative consequences of withdrawing incentives.

**Table 1:** Timeline of treatments.

Round	Date	Block 1	Block 2	
Baseline-I	Jul-14	Control (83)	Control (76)	
Baseline-II	Oct-14	Control (84)	Performance Pay (38)	Fixed Bonus (38)
Endline-I	Jan-15	Control (84)	Performance Pay (38)	
Endline-II	Apr-15	Control (84)		
Endline-III	Jul-15	Control (84)		

Notes: Numbers in parentheses show the number of centers in each arm. For Block 1 in the first baseline, one center was not surveyed as it was closed, thus 83 centers were surveyed instead of 84. Performance Pay is a bonus conditional on improvement in health outcomes promised at the end of Baseline-II and Endline-I. Payments were made at the end of Endline-I and Endline-II respectively. Fixed Bonus denotes a fixed bonus of Rs. 200 per worker at the end of Baseline-II.

The 84 centers in Block 1 served as a case-control group, to capture trends associated with seasonality or other shocks to child weights in Chandigarh as a whole. In the first round, we surveyed 83 centers from Block 1 as one center was temporarily closed. Incentive treatments were implemented in the 76 centers of Block 2, starting after the second baseline survey. Block 2 was chosen for the incentive treatments with an eye to external validity, because it had a lower average prevalence of malnutrition and hence a smaller fraction of the population susceptible to improvement. Previous studies suggested that lower prevalence raises the difficulty of detecting a statistically significant improvement (Singh, 2015; Singh and Mitra, 2017). Any significance of performance pay relative to the control group detected in Block 2 would therefore be more likely to hold elsewhere, improving the relevance of our study to other populations.

The 76 centers in Block 2 were randomly assigned to one of the two incentive treatments, through a lottery conducted in the workers’ presence. Half of the workers drew the performance pay treatment, and half drew the fixed bonus. The performance pay treatment payments were made first in February 2015 and then in May 2015. It is important to note here that an additional set of 85 ICDS centers were also part of this trial. They served as control sites for the first three rounds, after which they received tournament-type incentives in which caregivers compete against each other. Results for those treatments are reported in a separate study. This paper focuses on the contrast between performance payments and fixed bonuses in Block 2 relative to standard salaries in Blocks 1 and 3 for the first three rounds and standard salaries in Block 1 for the last two rounds.

Each administrative block is managed by a single officer and a set of field supervisors. Every supervisor is responsible for about 20 centers, each of which is staffed with one Anganwadi worker to serve about 30 preschool children. Monthly meetings of each block’s workers and supervisors are held with the block officer; this contributes to the homogeneity of information and conditions of service within each block, and required that our design provide treatments of similar value to workers within each block.

Table 2a shows summary statistics from the first baseline survey across our two treatment groups and the control block. Column 4 compares the performance pay arm in Block 2 to the pure control centers in Block 1, showing the intended differences with a lower prevalence of malnutrition (and hence a smaller population susceptible to improvement) in Block 2 as opposed to Block 1. To address differences between the two blocks, we stagger in statistical controls for observable characteristics that may differentiate Block 2 from Block 1, and focus on the differences between the randomly assigned treatments within Block 2. Column 5 provides a balance test between those two arms, showing that the only significant difference between them is in Panel C, as more workers are from scheduled castes or tribes in the performance pay treatment as opposed to the fixed-bonus arm. From panel A, we see that children in all centers have an average age of about 4.3 years and have roughly equal numbers of boys and girls, and that mothers are somewhat more likely than the workers to be from a scheduled caste or tribe. Mothers are much younger than the workers, and they have an average of two children in the home. Beyond these balance tests, our design includes two rounds of baseline surveys to test for any differences in pre-trends in outcomes and covariates that could threaten the assumption of common trends during the trial period. An Appendix provides details on these checks, starting with Table A1 in Supplementary material that gives us baseline correlations between health and individual covariates. This reveals that older children are taller and more likely to be underweight for their age and height. Malnutrition is also more widespread among children whose mothers are younger, illiterate and have lower income and assets, belonging to a scheduled caste, without a grandmother at home, and with more siblings at home. There is no significant effect of father’s literacy in this context, and having a toilet without a flush is correlated only with child height. Workers’ characteristics are generally not correlated with child health.

**Table 2a:** Summary Statistics at Baseline-I across treatment and control groups.

	Performance Pay	Fixed Bonus	Control	Performance Pay − Control	Performance Pay − Fixed Bonus
Panel A: Child and Household Characteristics					
Child's age	4.33 (0.93)	4.32 (0.94)	4.26 (0.90)	0.07 (0.05)	0.01 (0.06)
Gender (Male=1; Female=0)	0.48 (0.50)	0.50 (0.50)	0.50 (0.50)	−0.02 (0.01)	−0.01 (0.02)
Mother is SC/ST	0.64 (0.47)	0.66 (0.47)	0.59 (0.49)	0.06 (.03)*	−0.01 (0.04)
Mother is Hindu	0.90 (0.29)	0.92 (0.26)	0.90 (0.30)	0.01 (0.01)	−0.02 (0.02)
Mother's age	27.04 (3.58)	26.92 (3.32)	26.87 (3.74)	0.18 (0.17)	0.12 (0.18)
Grandmother at home	0.23 (0.42)	0.28 (0.43)	0.27 (0.45)	−0.04 (0.02)	−0.00 (0.03)
Total children in hh	2.01 (1.30)	1.99 (1.30)	2.28 (1.39)	−0.26 (0.06)***	0.02 (0.07)
Mother cannot read and write	0.27 (0.44)	0.25 (0.43)	0.39 (0.49)	−0.12 (0.03) ***	0.02 (0.03)
Husband cannot read and write	0.14 (0.35)	0.17 (0.38)	0.24 (0.43)	−0.09 (0.02) ***	−0.03 (0.03)
Mother is homemaker	0.79 (0.40)	0.77 (0.41)	0.78 (0.41)	0.01 (0.04)	0.02 (0.05)
Toilet communal	0.10 (0.29)	0.11 (0.31)	0.11 (0.31)	−0.02 (0.03)	−0.02 (0.04)
Toilet without flush	0.19 (0.39)	0.15 (0.35)	0.13 (0.34)	0.06 (0.04)	0.04 (0.06)
Fixed assets (out of 13)	0.52 (0.14)	0.54 (0.13)	0.47 (0.15)	0.05 (0.02) ***	−0.02 (0.02)

Panel B: Child Health					
Weight	13.67 (2.02)	13.48 (1.98)	13.27 (1.91)	0.40 (0.12) ***	0.20 (0.15)
Wfa Z-score	−1.50 (0.81)	−1.59 (0.83)	−1.65 (0.82)	0.14 (0.04) ***	0.08 (0.06)
Wfa grade	0.43 (0.63)	0.49 (0.67)	0.53 (0.70)	−0.11 (0.04) ***	−0.06 (0.05)
Wfa Malnutrition	0.36 (0.48)	0.39 (0.49)	0.41 (0.50)	−0.07 (0.03) **	−0.04 (0.04)
Height	97.21 (7.54)	97 (7.41)	96.88 (7.57)	0.34 (0.55)	0.23 (0.70)
Wfh Z-score	−0.78 (1.18)	−0.88 (1.14)	−1.05 (1.27)	0.27 (0.09) ***	0.10 (0.10)
Wfh grade	0.15 (0.42)	0.18 (0.43)	0.25 (0.53)	−0.10 (0.02) ***	−0.03 (0.03)
Wfh Malnutrition	0.13 (0.33)	0.16 (0.37)	0.20 (0.40)	−0.07 (0.02) ***	−0.03 (0.02)

Panel C: Worker and Anganwadi Characteristics					
Worker is SC/ST	0.52 (0.50)	0.21 (0.41)	0.45 (0.50)	0.07 (0.09)	0.30 (0.11) *
Worker is Hindu	0.83 (0.36)	0.84 (0.35)	0.87 (0.34)	−0.03 (0.07)	−0.01 (0.09)
Worker's age	41.21 (8.45)	39.11 (7.09)	38. 43 (8.16)	2.78 (1.55) *	2.10 (1.86)
Worker is college educated	0.26 (0.43)	0.40 (0.49)	0.37 (0.48)	−0.11 (0.08)	−0.14 (0.11)
Electricity in AWC	0.97 (0.16)	0.96 (0.18)	0.93 (0.26)	0.05 (0.04)	0.00 (0.04)
Fan in AWC	0.97 (0.16)	0.96 (0.18)	0.91 (0.28)	0.06 (0.04)	0.00 (0.04)
Drinking water in AWC	0.60 (0.48)	0.57 (0.49)	0.47 (0.50)	0.13 (0.02) ***	0.03 (0.03)
Toilet in AWC	0.56 (0.49)	0.56 (0.50)	0.46 (0.50)	0.10 (0.03) ***	0.01 (0.04)

Table 2b illustrates compliance and attrition rates across the three groups and five rounds. The total number of children weighed generally rises from round to round, from 4294 in the first baseline to 4550 in the last endline, reflecting the city’s overall population growth or an increase in attendance at the centers. There is a high degree of turnover at each center, with 20 percent or more of the children in each arm not being reweighed at the end of each three-month period. Attrition rates are lower during the periods of incentive treatments, indicating that caregivers are not selectively rejecting children whose weights have worsened, and that families may even be sending more children to the centers in response to improved services there. It is also notable that close to 90% of all mothers are surveyed in each round, which is helpful for identifying families’ responses to the caregivers’ actions. It is still possible that attrition is systematically different in the treatment and control groups. In particular, we would be concerned if those with higher weight for age z-scores were less likely to get weighed at midline and endline in the performance pay treatment group as in Jacob (2005). This could signal a change in the composition of the groups because of the treatment and we would need to correct for non-random attrition. Table A2 in Supplementary material columns (1) to (4) provide evidence that between rounds 2 and 3 (Baseline-II and Endline-I), there are no significant differential attrition rates and the attrition itself is not correlated with higher z-scores in the treatment groups. In the medium term, there is lower attrition in the performance pay group suggesting that the children are less likely to drop out or be absent from day care centers, with no evidence of non-random attrition across groups based on health.^2^

**Table 2b:** Compliance and attrition rates.

		Control	Performance Pay	Fixed Bonus	Total
Baseline 1	children weighed	2231	1028	1035	4294
	children whose mothers quizzed	1949	892	896	3737
	% children whose mothers quizzed	0.87	0.87	0.87	0.87

Baseline 2	children re-weighed	1526	809	800	3135
	children whose mothers re-quizzed	1270	645	640	2555
	Attrition in children weighed	0.32	0.21	0.23	0.27
	total children weighed (1)	2219	1073	1058	4350
	total children whose mothers quizzed (2)	1968	942	959	3869

Endline 1	children re-weighed from (1)	1753	855	835	3443
	children whose mothers re-quizzed from (2)	1451	701	700	2852
	Attrition in children weighed	0.21	0.20	0.21	0.21
	total children weighed (3)	2448	1096	1122	4666
	total children whose mothers quizzed (4)	2158	970	1009	4137

Endline 2	children re-weighed from (3)	1765	859	844	3468
	children whose mothers re-quizzed from (4)	1452	697	711	2860
	Attrition in children weighed	0.28	0.22	0.25	0.26
	total children weighed (5)	2328	1081	1117	4526
	total children whose mothers quizzed (6)	2099	933	982	4014

Endline 3	children re-weighed from (5)	1506	755	789	3050
	children whose mothers re-quizzed from (6)	1279	596	630	2505
	Attrition in children weighed	0.35	0.30	0.29	0.33
	total children weighed	2330	1081	1139	4550
	total children whose mothers quizzed	2075	949	976	4000

## Empirical specification and main results

The empirical specification for our main results is a standard difference-in-differences equation:

$$z_{ijt}-z_{ijt-1}=\alpha +\beta {\left(performance\right)}_{i}+\gamma {\left(fixed\right)}_{i}+\mu_{ijt-1}+\theta_{it-1}+\varepsilon_{ijt}$$

$$t\in \{\begin{array}{c} \left\{2\right\}pre-trends\\ \left\{3\right\}short-term\\ \left\{4\right\}medium-term\\ \left\{5\right\}long-term \end{array}$$

In the above equation the subscript, *t* represents the survey round, *i* is the caregiver or center, and *j* is the child. The main independent variables, *performance* and *fixed* take the value 1 if the child is in that treatment group and 0 otherwise. *β* and *γ* are our main coefficients of interest and they represent the impact of the two treatments. *μ_ijt_* is a term for the matrix of mother and child level control variables. *θ_it_* is center-level control variables. Heteroscedasticity-consistent errors, *ε_ijt_*, are clustered at the center level. Performance Pay was promised to workers in November 2014 based on individual weight-for-age targets and was paid out in February 2015. Another round of promises was made in February 2015 and payments were made in May 2015. Fixed bonus was an ex-ante incentive of Rs. 200 in November 2014.

All dependent variables are changes in a child's health status between two consecutive rounds. Weight is measured in kilograms. The z scores are calculated based on the WHO’s distribution of healthy weights in a well-nourished population at each age and sex, and malnutrition status is an indicator variable equal to one if the child’s weight is more than two standard deviations below the mean of the WHO’s healthy population at each age and sex. Given widespread stunting, in the Appendix we also report detailed data on changes in child height. Mother and child-level controls include age and sex of child, a dummy variables for if mother identifies herself as scheduled caste, if mother identifies herself as Hindu, if there is a grandmother at home, if mother cannot read and write, if husband cannot read and write, if mother is a homemaker, if toilet is communal, if toilet has no flush, mother's age, total children in household, household income and an index of 13 fixed assets in the household. Worker-level controls are dummy variables for if worker identifies herself as scheduled caste, if worker identifies herself as Hindu, if worker is college-educated, worker's age and dummy variables for the availability of the following resources at the center: electricity, fan, helper, chart, blackboard, drinking water and toilet.

Table 3 shows the effects on health outcomes after the introduction of treatments in the short-term, medium term and fading-out effects. All regressions are run with mother and child-level controls as well as worker-level controls. We find strongly significant effects of performance pay in the short term reflecting changes between Baseline-II and Endline-I, i.e., between October 2014 and January 2015 (columns (1)-(3)).^3^ With performance pay the average child gains about 220 g over three months relative to control. The difference is an increase of about 0.1 standard deviations relative to the distribution of healthy children’s weight-for-age, and a decline of 5.6 percentage points in the prevalence of weight-for-age malnutrition. The effect amounts to about 70 gms per month of treatment, which is comparable to the 100 gms per month achieved through deworming and iron supplementation to children of similar ages in Delhi’s slum areas (Bobonis et al., 2006). A community-based nutrition education program targeted towards mothers led to an increase in weight-for-age z-scores by about 0.16 standard deviations for children under-2 years in Ecuador as compared to 0.10 standard deviations here (Roche et al., 2016). The effects on child weight-for-age z-scores are also similar in magnitude to those found in Singh (2015) and Singh and Mitra (2017). The weight increase of 220 gms in the performance pay group is a roughly 40% increment above the control group’s gain of about 550 gms per child between the two rounds. The comparable coefficients on the fixed bonus treatment are about half as large and not statistically significant, with wide standard errors so we cannot reject them being different from the effect of the performance pay treatment.

**Table 3:** Effects on health outcomes after introduction of treatments.

	(1)	(2)	(3)	(4)	(5)	(6)	(7)	(8)	(9)
*change in Dependent Variable*	Weight	Wfa z	Wfa mal	Weight	Wfa z	Wfa mal	Weight	Wfa z	Wfa mal
	Short term			Medium term			Fading out effects		
Performance Pay	0.219^c^	0.101^c^	−0.0561^b^	0.231^c^	0.0976^c^	−0.0522^b^	0.0898	0.0355	−0.0338
	(0.0772)	(0.0370)	(0.0269)	(0.0687)	(0.0327)	(0.0219)	(0.0904)	(0.0408)	(0.0235)
Fixed Bonus	0.123	0.0557	−0.0333	0.196^b^	0.0878^b^	−0.0341	0.00967	0.00266	0.00262
	(0.0933)	(0.0442)	(0.0278)	(0.0776)	(0.0380)	(0.0241)	(0.0752)	(0.0357)	(0.0267)
p-value (P Pay − F Bonus)	0.2768	0.2829	0.4881	0.6336	0.7869	0.5092	0.3349	0.3894	0.1752
Mother and child-level controls	X	X	X	X	X	X	X	X	X
Worker-level controls	X	X	X	X	X	X	X	X	X
N	3528	3522	3524	2303	2301	2302	2230	2223	2224

Notes: Heteroscedasticity-consistent standard errors accounting for clustering at the center level in parentheses. Data are from two consecutive rounds of surveys carried out in October 2014 and January 2015. Performance Pay was promised to workers in November 2014 based on individual weight-for-age targets and was paid out in February 2015. Another round of promises was made in February 2015 and payments were made in May 2015. Fixed Bonus was an ex-ante incentive of Rs. 200 per worker in November 2014. All dependent variables are the changes in a child's health indicator over the two consecutive rounds. Weight is measured in kilograms. Wfa z is the weight-for-age z score given the child's sex and age. Wfh mal is an indicator for malnutrition as measured by weight-for-height z score and Wfa mal is an indicator based on weight-for-age z score. Mother and child-level controls include age and sex of child, a dummy variables for if mother identifies herself as scheduled caste, if mother identifies herself as Hindu, if there is a grandmother at home, if mother cannot read and write, if husband cannot read and write, if mother is a homemaker, if toilet is communal, if toilet has no flush, mother's age, total children in household, household income and an index of 13 fixed assets in the household. Worker-level controls are dummy variables for if worker identifies herself as scheduled caste, if worker identifies herself as Hindu, if worker is college-educated, worker's age and dummy variables for the availability of the following resources at the center: electricity, fan, helper, chart, blackboard, drinking water and toilet.

^a^Significant at 10%.

b Significant at 5%.

c Significant at 1%.

Columns (4)–(6) in Table 3 measure the subsequent medium-term impact of each treatment on our health outcomes, reflecting change from the first endline in January 2015 to the second endline in April 2015. We find that the short-term effects are sustained and significant, with performance pay again about the same magnitude of gains as in the previous three-month period. Controlling for observables results in somewhat similar-sized coefficients, which again indicate gains of about 230 g over three months, an increase of about 0.1 standard deviation in the weight-for-age z score, and a decline of about 5.2 percentage points in the prevalence of weight-for-age malnutrition. It is notable that workers who received the fixed bonus also achieved significant increases in child weights during this period, which again are not significantly different from the performance pay coefficients as depicted by the *p*-values of the differences between the two coefficients.

Columns (7)-(9) test for fading out or reversal of improvements in weight after discontinuation of treatments. Data refer to changes from the second endline in April 2015 to the third endline in July 2015. What we find is no further significant improvements but also no evidence of reversals to earlier malnutrition rates. Children in centers where caregivers had earlier received performance pay treatments experienced modest weight gains, improvements in z scores and reductions in malnutrition prevalence but these changes are not significantly different from changes in the control blocks, and coefficient estimates for children in centers which had the fixed bonus treatment are even closer to zero. This pattern suggests that performance pay works primarily as a direct incentive in this context, generating a one-time improvement without either entrenching or eroding the social norms and intrinsic motivations of the ICDS caregivers and the children’s own families.

**Fig. 2:**
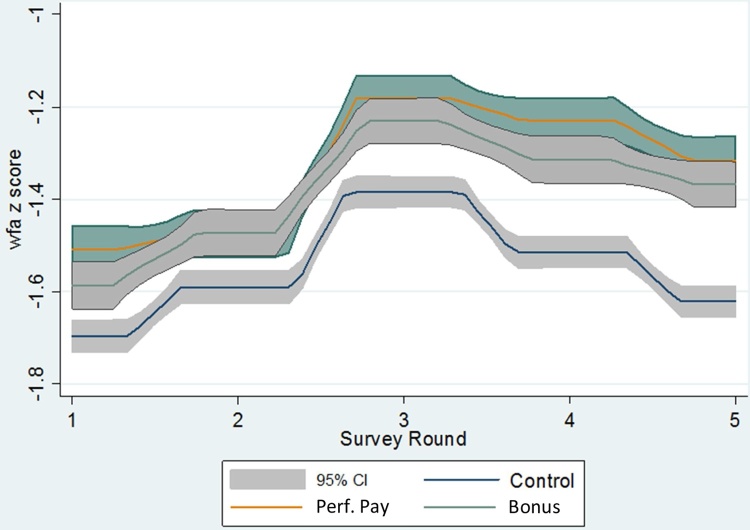
Weight-for-age z scores over five consecutive rounds between July 2014 and July 2015 in the two treatment groups and the control group.

For a visual representation of the unconditional results depicted in our tables, Fig. 2 shows the average weight-for-age Z-scores over the five consecutive rounds between July 2014 and July 2015 for the two treatment and control groups. The improvement from round 1–2 is a pre-trend that is shared by the control and fixed-bonus groups, with a smaller improvement in the performance-pay group; all groups continue from round 2–3, with a sharper increase in the performance-pay group to a higher level that persists in rounds 4 and 5, as the control group declines even faster than either treatment group. The factors involved in these common trends of initial improvement and then worsening in weight-for-age across all of our ICDS centers may involve fluctuations in real income and purchasing power, in addition to annual fluctuations in diet and disease associated with temperature and rainfall. Chandigarh is located in the far north of India, so temperatures and rainfall both declined sharply over the first two quarterly periods to their annual lows around January, and then rose again over the next two quarters to their annual peak temperatures in May-July and peak rainfall in June-September.

Our trial is designed around the specific focus of ICDS management and Indian policymakers on children’s malnutrition status, as measured by the prevalence of underweight. Nutritionists are also interested in stunting and attained height. Appendix Table A7 in Supplementary material tests for effects of the incentive treatments on children’s heights, in centimeters, between each successive survey round. This reveals a statistically significant increase of about one centimeter between R2 and R3, with no persistence or reversion in subsequent periods. This suggests that children’s linear growth was promoted by caregivers’ efforts in response to incentives at that time. The effects are robust to all controls, and are more significant and slightly larger for the performance-pay treatment than the fixed-bonus treatment, although again the difference between them is not significant.

## Robustness and heterogeneity

A first threat to our identification strategy is that the two treatment arms, which have a lower initial prevalence of malnutrition in the first baseline survey, also have faster trend improvements over time. To test for this we repeated the baseline survey. Results are reported in Table A8 in Supplementary material, showing no pre-trend differences in the performance pay arm relative to the control group; in the fixed bonus arm, weight for age z scores improve slightly faster with significance only at the 10% level, but that effect is eliminated by controlling for observable differences as we do in the main regressions. From this we conclude that pre-trend differences cannot have contributed to the significant effect we found for performance pay.

The main effect we find is the immediate impact of performance pay over the three month period from October 2014 to January 2015. Table 4 tests for heterogeneity of this effect, splitting the sample by gender (columns (1) and (2)), age (columns (3)-(5)), literacy of parents (column (6)-(9)), total children in household (columns (10) and (11)), and fixed assets owned by household (columns (12) and (13)). We find that the performance pay treatment has a similar effect size across all these subgroups; the effect remains statistically significant in all except the small sub-sample of children whose fathers are illiterate. The smaller effect of the fixed bonus treatment is significant only among boys and younger children, in columns (1) and (3). However, the only significant differences between the short-term impacts of the performance pay and fixed bonus treatments are for girls and illiterate mothers. Here, the *p*-values of the differences between the coefficients are equal to about 0.02.^4^

**Table 4:** Heterogeneity check for short term effects.

	Boys	Girls	3–4 years	4–5 years	5–6 years	Literate mother	Illiterate mother	Literate father	Illiterate father	Total children < = 2	Total children >2	Fixed assets > median	Fixed assets < median
	(1)	(2)	(3)	(4)	(5)	(6)	(7)	(8)	(9)	(10)	(11)	(12)	(13)
*change in Dependent Variable*	Weight	Weight	Weight	Weight	Weight	Weight	Weight	Weight	Weight	Weight	Weight	Weight	Weight
Performance Pay	0.202^c^	0.264^c^	0.257^c^	0.212^b^	0.277^b^	0.203^c^	0.297^c^	0.236^c^	0.172	0.266^c^	0.190^b^	0.200^b^	0.234^c^
	(0.0680)	(0.0812)	(0.0759)	(0.104)	(0.112)	(0.0753)	(0.0972)	(0.0727)	(0.133)	(0.0713)	(0.0835)	(0.0906)	(0.0866)
Fixed Bonus	0.201^b^	0.0102	0.145^a^	0.0820	0.0668	0.123	0.0348	0.0874	0.114	0.134	0.0690	0.0498	0.126
	(0.0886)	(0.0939)	(0.0865)	(0.106)	(0.141)	(0.0998)	(0.102)	(0.0877)	(0.182)	(0.0823)	(0.0944)	(0.105)	(0.107)
p-value (P Pay − F Bonus)	0.9907	0.0202	0.2655	0.2728	0.1768	0.4342	0.0286	0.1154	0.7766	0.1476	0.2251	0.1512	0.3861
N	2545	2650	2005	1807	1175	2729	1524	3239	977	2802	2401	1904	2442

Notes: Heteroscedasticity-consistent standard errors accounting for clustering at the center level in parentheses. Data are from two consecutive rounds of surveys carried out in October 2014 and January 2015. Performance Pay was promised to workers in November 2014 based on individual weight-for-age targets and was paid out in February 2015. Fixed Bonus was an ex-ante incentive of Rs. 200 per worker in November 2014. All dependent variables are the changes in a child's health indicator over the two consecutive rounds. Weight is measured in kilograms. Columns (1) and (2) split the sample by sex of child, columns (3)-(5) by age of child, column (6)-(9) by literacy of parents, columns (9) and (10) by total children in household less than or greater than the median of 2, and columns (12) and (13) by the median proportion of 13 fixed assets owned by household (46%).

a Significant at 10%.

b Significant at 5%.

c Significant at 1%.

An important test for the incentive effect of performance pay is to test for threshold effects. Payments are based on the number of children in each malnutrition category, which provides a greater incentive per unit of weight gain in children who are closer to the threshold and more likely to move up to the next category. Focusing efforts on children near the threshold is not necessarily desirable, since it may lead to others being neglected (as in Neal and Schanzenbach, 2010). We define “near” the threshold as a child being closer to the next category than the median child in their category. In Table 5, among moderately malnourished children in columns (1) and (2), we find that weight-for-age z-scores increase significantly more in the performance pay treatment than in the fixed bonus treatment for those nearer the threshold, with no such effects among those far from the threshold. This is consistent with our conceptual framework. Among the much smaller set of severely malnourished children, in columns (3) and (4), we find no significant effects perhaps due to low sample size. Among the large number of normal weight children, in columns (5) and (6), we find that all experience roughly similar benefits from treatment in the sense of avoiding declines into the moderately malnourished category, with no significant differences between the two treatments.^5^

**Table 5:** Threshold effects.

	Near	Far	Near	Far	Near	Far
	Moderate		Severe		Normal	
	(1)	(2)	(3)	(4)	(5)	(6)
*change in Dependent Variable*	Wfa z	Wfa z	Wfa z	Wfa z	Wfa z	Wfa z
Performance Pay	0.142^c^	0.0689	0.101	0.0659	0.138^c^	0.199^c^
	(0.0472)	(0.0521)	(0.121)	(0.142)	(0.0409)	(0.0432)
Fixed Bonus	0.0795^a^	0.109	0.0297	−0.0293	0.0843^b^	0.121^b^
	(0.0479)	(0.0783)	(0.0977)	(0.165)	(0.0414)	(0.0512)
p-value (P Pay − F Bonus)	0.3051	0.6371	0.5902	0.6237	0.2491	0.1586
N	710	767	250	224	1650	1568

Notes: Heteroscedasticity-consistent standard errors accounting for clustering at the center level in parentheses. Data are from two consecutive rounds of surveys carried out in October 2014 and January 2015. Performance Pay was promised to workers in November 2014 based on individual weight-for-age targets and was paid out in February 2015. Fixed Bonus was an ex-ante incentive of Rs. 200 per worker in November 2014. Near means that in Round 2, child was closer to the target weight than the median difference between present weight and target weight for a child in the Moderate category (within 1 kg of the Normal target), Severe category (within 2.5 kg of the Moderate target), and Normal category (within 0.900 kg of the Moderate threshold). Far is defined as not Near.

a Significant at 10%.

b Significant at 5%.

c Significant at 1%.

Next, in Table 6, we investigate the cost function for improvement by testing for heterogeneity based on initial prevalence of malnutrition. Having a larger number of children classified as malnourished ensures that a given effort could generate a larger number of improvements in status, but also signals the presence of obstacles to improvement such as worse sanitation and greater disease transmission, poorer diets at home, greater poverty, lower parental or worker knowledge. We use two criteria to classify centers at baseline, first by the absolute number of malnourished children relative to the median value of 10 children per center, and then by the proportion of children who were malnourished relative to the median value of 0.36. In the short term over the first three months we find no significant differences in treatment effects between centers with high and low malnutrition at baseline, but over the subsequent three months there are more improvements in centers with higher initial prevalence. This suggests that increased efforts may eventually be more cost-effective in situations with worse initial conditions, when caregivers invest in sustained efforts of the type incentivized by performance pay.

**Table 6:** Dose Response effects.

	Low #mal	High #mal	Low malp	High malp	Low #mal	High #mal	Low malp	High malp
	Short-term		Short-term		Medium-term		Medium-term	
	(1)	(2)	(3)	(4)	(5)	(6)	(1)	(2)
*change in Dependent Variable*	Wfa z	Wfa z	Wfa z	Wfa z	Wfa z	Wfa z	Wfa z	Wfa z
Performance Pay	0.119^c^	0.140^c^	0.149^c^	0.138^c^	0.0404	0.0751^b^	0.0411	0.0874^c^
	(0.0311)	(0.0525)	(0.0382)	(0.0435)	(0.0406)	(0.0315)	(0.0440)	(0.0318)
Fixed Bonus	0.0338	0.0865^a^	0.0764	0.0625	0.0714	0.0378	0.0766	0.0373
	(0.0399)	(0.0518)	(0.0465)	(0.0502)	(0.0505)	(0.0380)	(0.0556)	(0.0350)
p-value (P Pay − F Bonus)	0.0399	0.3751	0.1376	0.1809	0.5065	0.363	0.4646	0.1928
N	3014	2155	2636	2533	1607	1829	1482	1954

Notes: Heteroscedasticity-consistent standard errors accounting for clustering at the center level in parentheses. Data are from two consecutive rounds of surveys carried out in October 2014 and January 2015. Performance Pay was promised to workers in November 2014 based on individual weight-for-age targets and was paid out in February 2015. The performance pay was then promised again to the same set of workers in February 2015 and paid out in May 2015. Fixed Bonus was an ex-ante incentive of Rs. 200 per worker in November 2014. Medium-term is measurement between January 2015 and April 2015. Low #mal is the subset of centers where total malnourished children at baseline were less than or equal to the median (10 children). Low malp is defined as the subset of centers where the proportion of malnourished children at baseline were less than or equal to the median (0.36). High #mal and High malp are the centers that are not Low #mal and Low malp respectively.

a Significant at 10%.

b Significant at 5%.

c Significant at 1%.

## Mechanisms

The specific kinds of effort undertaken by caregivers in response to incentives are shown in Table 7, by testing for treatment effects on each category of worker-mother interaction: home visits by worker, center visits by mother, and frequency of worker talking about child. Each estimate refers to actions by the caregiver in the last month as reported independently by mothers. We find large and significant effects from both treatments on the frequency of worker talking about child in the short run (column 3), followed by similarly large and significant effects from both treatments on the frequency of home visits in the medium run (column 6) and then also center visits by the mother and frequent talking about the child after incentives ended (columns 8 and 9) especially under performance pay. This could reflect a typical sequence of actions by which workers connect with mothers, with higher quality service under performance pay eliciting greater response by the mother.

**Table 7:** Mechanism − Quantity of interaction between worker and mother.

	Short-term			Medium-term			After incentives ended		
	(1)	(2)	(3)	(4)	(5)	(6)	(7)	(8)	(9)
*change in Dependent Variable*	Home visits by worker	Center visits by mother	Frequency of worker talking about child	Home visits by worker	Center visits by mother	Frequency of worker talking about child	Home visits by worker	Center visits by mother	Frequency of worker talking about child
Performance Pay	−1.256	−1.141	4.410^c^	4.434^c^	−1.468	0.834	1.172	2.798^b^	2.637^c^
	(0.915)	(1.438)	(0.970)	(0.869)	(1.229)	(1.323)	(0.824)	(1.236)	(0.921)
Fixed Bonus	−2.019^a^	−1.223	5.012^c^	4.570^c^	−0.841	1.876	1.327	2.618^a^	1.803^a^
	(1.092)	(0.855)	(1.029)	(0.929)	(1.248)	(1.354)	(0.886)	(1.411)	(1.009)
p-value (P Pay − F Bonus)	0.8535	0.6478	0.4784	0.8535	0.6478	0.4784	0.8786	0.884	0.489
Mother and child-level controls	X	X	X	X	X	X	X	X	X
Worker-level controls	X	X	X	X	X	X	X	X	X
N	3275	2831	3062	2108	1753	1997	2108	1881	1977

Notes: Heteroscedasticity-consistent standard errors accounting for clustering at the center level in parentheses. Data are from four quarterly consecutive rounds of surveys carried out between October 2014 and July 2015. Performance Pay was promised to workers in November 2014 based on individual weight-for-age targets and was paid out in February 2015. Fixed Bonus was an ex-ante incentive of Rs. 200 per worker in November 2014. All dependent variables are the changes in the indicator over the two consecutive rounds. Home visits by worker are number of visits by Anganwadi worker to the household in the previous month as reported by the mother. Center visits by mother are mother's visits to the Anganwadi in the previous month as reported by the mother. Frequency of worker talking about child is the number of times worker spoke about child with mother in the previous month as reported by the mother. Mother and child-level controls include age and sex of child, a dummy variables for if mother identifies herself as scheduled caste, if mother identifies herself as Hindu, if there is a grandmother at home, if mother cannot read and write, if husband cannot read and write, if mother is a homemaker, if toilet is communal, if toilet has no flush, mother's age, total children in household, household income and an index of 13 fixed assets in the household. Worker- level controls are dummy variables for if worker identifies herself as scheduled caste, if worker identifies herself as Hindu, if worker is college-educated, worker's age and dummy variables for the availability of the following resources at the center: electricity, fan, helper, chart, blackboard, drinking water and toilet.

a Significant at 10%.

b Significant at 5%.

c Significant at 1%.

The content of communication between workers and mothers is addressed in Table 8, obtained by asking each mother what the caregiver talked about during one or more conversations over the previous month in the initial period of incentive treatments. Each is a dummy variable equal to 1, if the worker spoke to the mother about her child’s nutrition, about her child’s hygiene, showed the child’s growth chart, or scared the mother about consequences of malnutrition. We find that in the short run both treatments increased communication only about nutrition (column 1), perhaps in part because that was facilitated by the availability of recipe books. In the medium run they reverted to less communication about nutrition and also about growth charts, with more communication about hygiene in the fixed bonus arm (columns 5–7), and then increased communication about all three of these topics in both arms after incentives end (columns 9–12). Again this sequence of adjustments could reflect the evolution of topics addressed by workers, as they learn about how mothers and children respond to their efforts.

**Table 8:** Mechanism − Quality of information provided by worker to mother.

	Short-term				Medium-term				After incentives ended			
	(1)	(2)	(3)	(4)	(5)	(6)	(7)	(8)	(9)	(10)	(11)	(12)
*change in Dependent Variable*	Nutrition	Hygiene	Chart	Scare	Nutrition	Hygiene	Chart	Scare	Nutrition	Hygiene	Chart	Scare
Performance Pay	0.226^c^	0.0949	0.0712	−0.0206	−0.306^c^	0.0843	−0.341^c^	−0.133	0.316^c^	0.313^c^	0.440^c^	0.0721
	(0.0767)	(0.0832)	(0.0780)	(0.0866)	(0.0802)	(0.0805)	(0.105)	(0.0876)	(0.0830)	(0.0896)	(0.120)	(0.0594)
Fixed Bonus	0.245^c^	0.0757	0.0138	−0.0922	−0.177^b^	0.246^c^	−0.487^c^	−0.0122	0.265^c^	0.207^c^	0.449^c^	−0.0160
	(0.0633)	(0.0907)	(0.0792)	(0.0725)	(0.0747)	(0.0795)	(0.0981)	(0.0916)	(0.0837)	(0.0748)	(0.113)	(0.0705)
p-value (P Pay − F Bonus)	0.8465	0.8727	0.5659	0.5144	0.1514	0.1096	0.2273	0.2205	0.5252	0.3086	0.9428	0.2705
Mother and child-level controls	X	X	X	X	X	X	X	X	X	X	X	X
Worker-level controls	X	X	X	X	X	X	X	X	X	X	X	X
N	3223	3223	3223	3223	2024	2024	2024	2024	2034	2034	2034	2034

Notes: Heteroscedasticity-consistent standard errors accounting for clustering at the center level in parentheses. Data are from four quarterly consecutive rounds of surveys carried out between October 2014 and July 2015. Performance Pay was promised to workers in November 2014 based on individual weight-for-age targets and was paid out in February 2015. Fixed Bonus was an ex-ante incentive of Rs. 200 per worker in November 2014. All dependent variables are the changes in an indicator over the two consecutive rounds. Nutrition is a dummy variable equal to 1 if, in the last month, the worker spoke to the mother about her child's nutrition. Hygiene is a dummy variable equal to 1 if, in the last month, the worker talked with the mother about maintaining child's hygiene. Chart is a dummy variable equal to 1 if, in the last month, the worker showed the mother a growth chart. Scare is a dummy variable equal to one if, in the last month, the worker scared the mother with consequences of malnutrition. Mother and child-level controls include age and sex of child, a dummy variables for if mother identifies herself as scheduled caste, if mother identifies herself as Hindu, if there is a grandmother at home, if mother cannot read and write, if husband cannot read and write, if mother is a homemaker, if toilet is communal, if toilet has no flush, mother's age, total children in household, household income and an index of 13 fixed assets in the household. Worker-level controls are dummy variables for if worker identifies herself as scheduled caste, if worker identifies herself as Hindu, if worker is college-educated, worker's age and dummy variables for the availability of the following resources at the center: electricity, fan, helper, chart, blackboard, drinking water and toilet.

^a^Significant at 10%.

b Significant at 5%.

c Significant at 1%.

Next we address families’ response to caregivers’ efforts in Table 9. The dependent variable is based on mothers’ reported dietary intake for her child at home, focusing on four foods of particular nutritional interest: milk and green vegetables for micronutrients, and traditional desserts or porridge for calories. These are dummy variables, coded 1 if the mother reports feeding these at least twice in a week on average. In Table 9 we find significant and large positive effects on milk, dessert and porridge consumption in the short term in both treatment groups, at the expense of a negative effect on green vegetablesn both treatment groups, which could be due to substitution among these foods or in response to the foods children have eaten at the ICDS center. In the medium term, there is a further positive effect for milk in both arms, followed by some reversion after incentives end.^6^

**Table 9:** Mechanism − Diet at home.

	Short-term				Medium-term				After incentives ended			
	(1)	(2)	(3)	(4)	(5)	(6)	(7)	(8)	(9)	(10)	(11)	(12)
*change in Dependent Variable*	Milk	Green veg	Dessert	Porridge	Milk	Green veg	Dessert	Porridge	Milk	Green veg	Dessert	Porridge
Performance Pay	0.0557^b^	−0.100^c^	0.207^c^	0.108^a^	0.125^c^	−0.0212	−0.116^a^	0.169^b^	−0.0819^c^	−0.0460	−0.0430	−0.111^b^
	(0.0217)	(0.0325)	(0.0559)	(0.0642)	(0.0317)	(0.0430)	(0.0618)	(0.0657)	(0.0262)	(0.0554)	(0.0697)	(0.0544)
Fixed Bonus	0.0608^c^	−0.150^c^	0.184^c^	0.289^c^	0.0902^c^	−0.0462	−0.0867	−0.0338	−0.0560^b^	−0.0946^c^	−0.0101	−0.0131
	(0.0205)	(0.0301)	(0.0561)	(0.0581)	(0.0284)	(0.0452)	(0.0673)	(0.0708)	(0.0271)	(0.0354)	(0.0635)	(0.0584)
p-value (P Pay − F Bonus)	0.8402	0.1681	0.7361	0.025	0.2124	0.4676	0.6948	0.011	0.151	0.3191	0.6381	0.1071
Mother and child-level controls	X	X	X	X	X	X	X	X	X	X	X	X
Worker-level controls	X	X	X	X	X	X	X	X	X	X	X	X
N	2300	2297	2268	2276	1517	1518	1505	1508	1433	1432	1419	1416

Notes: Heteroscedasticity-consistent standard errors accounting for clustering at the center level in parentheses. Data are from four quarterly consecutive rounds of surveys carried out between October 2014 and July 2015. Performance Pay was promised to workers in November 2014 based on individual weight-for-age targets and was paid out in February 2015. Fixed Bonus was an ex-ante incentive of Rs. 200 per worker in November 2014. All dependent variables are the changes in an indicator over the two consecutive rounds. Milk, Green veg, Dessert (traditional) and Porridge are dummy variables equal to 1 if the mother reports feeding these items at least twice a week to her child. Mother and child-level controls include age and sex of child, a dummy variables for if mother identifies herself as scheduled caste, if mother identifies herself as Hindu, if there is a grandmother at home, if mother cannot read and write, if husband cannot read and write, if mother is a homemaker, if toilet is communal, if toilet has no flush, mother's age, total children in household, household income and an index of 13 fixed assets in the household. Worker-level controls are dummy variables for if worker identifies herself as scheduled caste, if worker identifies herself as Hindu, if worker is college-educated, worker's age and dummy variables for the availability of the following resources at the center: electricity, fan, helper, chart, blackboard, drinking water and toilet.

a Significant at 10%.

b Significant at 5%.

c Significant at 1%.

Our annex of supplemental information includes detailed results for other possible mechanisms, such as whether incentivized workers are able to avoid declines in status among normal weight children, with and without statistical controls. Table A15 in Supplementary material lists the increases and declines in the short and medium-term across the treatment and control arms. In line with our intuition we find that performance pay treatment acts both on reducing malnutrition as well as on not allowing worsening of normal weight children. The table also shows that 20 percent of the children are in a state of flux between rounds and the weights are not as persistent as expected even in the control group. For example, moving from winters (January 2015) to summer (April 2015), appears to worsen 13% of the normal weight children in the control group. This points to seasonality in weights, and lower vulnerability during the summer for those affected by the performance pay and fixed bonus treatments. Future work in determining the magnitude of weight-for-age malnutrition in a region should consider the impact of the season and countervailing factors during seasonality. For instance, in Figures A1 and A2 in supplementary material, we note that among the various correlates of weight-for-age malnutrition, sanitation practices appear to be more strongly negatively correlated during onset of summer. Not having a flush toilet is correlated with lower weight-for-age in April 2015 but not in January 2015.^7^

A final mechanism check reported in the annex concerns absenteeism during the months between rounds of data collection. Independent supervisors were hired to make unannounced visits to the centers in August-September 2014, November-December 2014, February-March 2015 and May-June 2015, to check for presence of the worker and to weigh the food served at the center as well. Table A17 in supplementary material shows that the attendance of the workers went up significantly in the performance pay treatment in the month of November after the incentives were promised to them. There are also sporadic increases in attendance among workers who received the fixed bonus treatment, but the timing of these increases cannot be clearly linked to payment dates so may be due to random fluctuations in absenteeism. Overall, there is a significant increase in attendance of Anganwadi workers in the performance pay treatment in the short-term. We also show the increases graphically in the three groups in Appendix Figures A5, A6 and A7 in Supplementary material. However, we do not find any evidence to suggest that the weight of the total food being served increased in the performance pay or fixed bonus arm.

## Conclusion

This paper describes a randomized controlled trial of financial incentives for improved service delivery among 160 government workers in ICDS centers caring for over 4000 children in Chandigarh, India. In this trial, workers in centers selected for treatment randomly drew either a one-time fixed bonus of Rs. 200, or a performance-pay incentive of Rs. 200 per child at their center whose classification improved from severe to moderate or moderate to no malnutrition, net of any children whose classification worsened, over two successive three-month periods. This criterion for performance pay directly reflected the government’s goal to reduce prevalence of underweight in ICDS centers, as measured by each child’s weight relative to a healthy population at each age and sex. Workers on performance pay contracts had high expectations from themselves, as more than 50% of the workers expected to receive the maximum incentive possible (annex Figure A8). Their expectations were highly optimistic. Overall, the average payouts in the performance pay treatments were close to Rs. 600, less than half that of their mean expectation (annex Figure A9). Payouts are also somewhat smaller than the value per hour of the increased worker attendance we actually observe, suggesting an important role for voluntary efforts and intrinsic motivation.^8^

Our trial compared outcomes in the two treatment arms with each other, and with children at case-control centers in another part of Chandigarh. All children attending every center in the trial were measured on five successive occasions, through two baseline surveys to detect any trend differences prior to the trial, and then three endline surveys to detect short- and medium-term responses to treatment followed by persistence or reversal after incentives are removed. Surveys also included interviews with mothers about their interactions with the ICDS caregiver, and about what their child ate at home. Unannounced visits to each center in between the surveys were used to monitor caregiver effort.

Our principal finding is that workers receiving performance pay achieved significant improvements in children’s weights, averaging an increase of about 200 g per child relative to control, weight-for-age z score improvement of 0.1 standard deviations, and reduction in the prevalence of malnutrition by 5 percentage points over the first three months of performance pay. In the short-term, the number of malnourished children in a center declined by an average of 2 in the performance pay group and by 1 in the fixed bonus group relative to the control group change (of improving 2 children). Similar improvements were achieved in the performance pay group over the second three months of performance pay. Our robustness checks find no differences in pre-treatment time trends between arms of the trial, and mechanism tests reveal significant increases in the frequency with which caregivers receiving performance pay actually discussed nutrition with mothers, and significant increases in the frequency with which those mothers reported feeding milk, porridge and desserts to their child.

Improvements were also observed among children in centers where workers received the fixed bonus, implying that unconditional rewards can also elicit increased efforts. Performance pay had larger and more precisely estimated effects, especially for children closer to malnutrition thresholds. Performance pay was also more expensive and difficult to provide, however, so policymakers might prefer fixed payments designed to enhance workers’ intrinsic motivations.

The trial reported in this study builds on Singh (2015), continuing a series of trials designed to inform performance pay in the ICDS system in India. Related research concerns the use of tournament-type contests among Anganwadi workers in ICDS centers, the specific kinds of effort that workers use to achieve children’s weight gain, and complementarity or substitution between what they provide and children’s diets or care practices at home. Replication of this trial will be needed to confirm its validity, but results to date provide grounds for optimism that low-cost incentives can help public service providers significantly improve child health outcomes.

## Footnotes

1 An early example is Berry and Kanouse (1987), who found that physicians were more likely to complete and return a long questionnaire when paid an unconditional honorarium before doing so, rather than a conditional honorarium afterwards. Fehr and Gachter (2001) find that workers put in less effort when threatened with fines and that they do not respond reciprocally to higher offered wages in the presence of threats. Charness et al. (2004) find evidence of less gift exchange when subjects are shown explicit payoff table giving net profits for all wages and effort levels.

2 Table A3 in Supplementary material provides the unconditional means for each weight indicator across rounds and treatments. In particular, from the second baseline (round 2) to the first and second endlines (rounds 3 and 4) we find a striking improvement in both treatment arms of children’s average weight, weight-for-age z score and prevalence of weight-for-age malnutrition. Hypothesis tests to identify the significance and magnitude of these changes are detailed in the main results.

3 Tables A4, A5 and A6 show the short-term, medium-term and fading-out effects of the two treatments on each health outcome with fewer controls. In each of these tables, Columns (1) − (3) show unconditional changes in each of the main health indicators namely weight, weight-for-age z score (Wfa z), and weight-for-age malnutrition (Wfa mal) in the two treatment groups, relative to the control group without any additional control variables. Columns (4)-(6) add mother and child controls. In all these tables, results are consistent with adding in all controls.

4 The second effect we find is medium-term persistence of performance pay when continued from January to April 2015. Table A9 in Supplementary material shows that the magnitude and significance of this effect is generally robust across sub-samples.

5 Of late, bound estimators have been proposed to counter non-random attrition and these require relatively few assumptions. These estimators determine an interval for the true treatment effect based on extreme assumptions about the impact of selection on estimated effect that are consistent with the data. We carry out estimation of Lee (2009) bounds on our treatment effects to understand the range of estimates in the short-term and medium-term with assumptions about non-random attrition in one direction or another. This corresponds to two extreme assumptions about missing information that are consistent with the observed data and a one-sided selection mechanism (Tauchmann, 2013). In the group that suffers less from attrition, either the largest or the smallest values of the outcome are excluded from analysis.

6 In the Appendix Table A13 in Supplementary material we test for pre-trends in the mechanisms of quantity and quality of information and diet. We find differential time trends in only two of 22 comparisons, one of which is significant only at the 10% level, from which we conclude that the common trends assumption is likely to hold even for the mechanism checks. Table A14 in Supplementary material gives us performance pay treatment effect Lee (2009) bounds of between 0.057 and 0.388 for the short term and between 0.050 and 0.275 for the medium term. In both cases, the upper bound is significant at the 1% significance level. The upper bounds for the fixed bonus treatment are lower than the performance pay treatment in both the short and medium-term. Even though the bounds themselves are less precisely estimated for the fixed bonus treatment in the medium term, the lower and upper bound are close to one another suggesting that there was a medium term gain in the fixed bonus treatment that cannot be explained away by attrition.

7 As the treatment varies at the center level, there could be serial correlation in the health of children within a center that should be taken into account when estimating standard errors. One way of correcting for this issue is to follow a non-parametric approach and cluster standard errors at the center level (as we do in the earlier tables). Another recommendation is to carry out a Moulton (1986) parametric correction for the standard errors to allow for serial correlation and check for consistency of the main results. We do so in Table A16 in Supplementary material and find that our results remain robust.

8 This comparison is based on monthly earnings of Rs. 4000 per month, which values workers’ time at about Rs. 50 per hour for 20 days of 4 working hours each day. We find that performance pay increases worker attendance from 70 to 93 percent of our unannounced visits, implying an average increase from 14.0 to 18.6 days per month of actual work, or about 18 additional hours for a maximum cost of Rs. 900 in additional time spent over in their first month’s response to the incentive scheme, relative to average performance pay of Rs 600 over three months.
