# Supplementary material for: Impact of caregiver incentives on child health: Evidence from an experiment with Anganwadi workers in India
Source: J Health Econ. 2017 Sep;55:219–31. doi: 10.1016/j.jhealeco.2017.07.005 (PMC5597043; doi:10.1016/j.jhealeco.2017.07.005)
Supplement: Supplementary file 1 [file mmc1.docx]

**APPENDIX - TABLES**

Age of child

Table A1: Baseline correlations between health and individual covariates

(1)

Weight

(2)

Wfa z-score

(3)

Wfh mal

(4)

Wfa mal

(5)

Height

1.109***

(0.0323)

-0.206***

(0.0158)

0.0256***

(0.00819)

0.0884***

(0.00856)

4.851***

(0.132)

Sex of Child (Male=1, Female=0)

0.440***

(0.0446)

-0.0132

(0.0221)

0.0287**

(0.0116)

-0.00982

(0.0134)

0.877***

(0.172)

Mother is SC

-0.151***

(0.0575)

-0.0743***

(0.0279)

0.00236

(0.0124)

0.0405**

(0.0159)

-0.475**

(0.201)

Mother is Hindu

0.0611

(0.115)

0.0267

(0.0582)

-0.0199

(0.0252)

-0.0203

(0.0290)

-0.106

(0.346)

Mother's age

0.0304***

(0.00824)

0.0154***

(0.00402)

-0.00105

(0.00180)

-0.00589**

(0.00228)

0.0940***

(0.0294)

Grandmother is home

0.142**

(0.0563)

0.0750***

(0.0277)

-0.00244

(0.0147)

-0.0450***

(0.0156)

0.809***

(0.236)

Total children in hh

-0.109***

(0.0253)

-0.0543***

(0.0123)

-0.00274

(0.00622)

0.0270***

(0.00707)

-0.377***

(0.0881)

Total hh income ('0000)

0.131*

(0.0771)

0.0696*

(0.0376)

0.0157

(0.0202)

-0.0306

(0.0231)

0.853***

(0.309)

Mother is illiterate

-0.136**

(0.0609)

-0.0685**

(0.0297)

-0.00118

(0.0143)

0.0193

(0.0174)

-0.409**

(0.206)

Father is illiterate

-0.00597

(0.0658)

-0.00907

(0.0328)

-0.000306

(0.0164)

0.0303

(0.0191)

-0.00916

(0.249)

Mother is homemaker

-0.0269

(0.0649)

-0.00969

(0.0305)

0.0285*

(0.0155)

0.0120

(0.0188)

0.427

(0.266)

Toilet is communal

0.0842

(0.102)

0.0515

(0.0499)

0.0233

(0.0257)

-0.0156

(0.0289)

-0.0764

(0.432)

Toilet has no flush

-0.0510

(0.112)

-0.00859

(0.0563)

-0.0536

(0.0355)

0.0379

(0.0349)

-0.890**

(0.351)

Fixed assets Index

0.488**

(0.230)

0.234**

(0.110)

0.0584

(0.0501)

-0.126*

(0.0654)

2.402***

(0.840)

Worker is SC

-0.0155

(0.0754)

-0.00638

(0.0368)

-0.000291

(0.0191)

-0.00513

(0.0202)

-0.247

(0.312)

Worker is Hindu

0.00568

(0.121)

0.00744

(0.0589)

0.0584**

(0.0272)

-0.00619

(0.0334)

1.152**

(0.501)

Worker's age

0.00439

(0.00507)

0.00250

(0.00246)

0.000640

(0.00132)

-0.00166

(0.00139)

-0.00417

(0.0242)

Worker is College educated

0.0779

(0.0808)

0.0385

(0.0399)

-0.0268

(0.0209)

-0.00830

(0.0228)

-0.352

(0.333)

N

adj. R-sq

4942

0.297

4926

0.065

4674

0.010

4938

0.037

4935

0.366

Notes: Heteroscedasticity-consistent

standard errors accounting for clustering at the center level in

parentheses. Data are from the Baseline survey carried out in July 2014. Weight is measured in

kilograms. Wfa z is the weight-for-age z score given the child's sex and age. Wfh mal is an indicator for malnutrition as measured by weight-for-height z score and Wfa mal is an indicator based on weight-for- age z score. Mother and child-level controls include age and sex of child, a dum my variables for if mother identifies herself as scheduled caste, if mother identifies herself as Hindu, if there is a grandmother at home, if mother cannot read and write, if husband cannot read and write, if mother is a homemaker, if toilet is communal, if toilet has no flush, mother's age, total children in household, household income and an index of 13 fixed assets in the household. Worker-level controls are dummy variables for if worker identifies herself as scheduled caste, if worker identifies herself as Hindu, if worker is college-educated, worker's age and center's infrastructure. *Significant at 10%, **Significant

at 5%, ***Significant at 1%.

Table A2: Differential Attrition by Child Health between Rounds 2 and 4

Between Round 2 and Round 3

Between Round 3 and Round 4

(1)

Attrited

(2)

Attrited

(3)

Attrited

(4)

Attrited

(5)

Attrited

(6)

Attrited

(7)

Attrited

(8)

Attrited

Wfa z-score

-0.00400

(0.00827)

0.0121

(0.00849)

0.00986

(0.00902)

0.0109

(0.00886)

-0.0180*

(0.00980)

-0.00358

(0.00985)

-0.00610

(0.0118)

-0.00641

(0.0116)

Performance Pay

-0.0111

(0.0400)

-0.0167

(0.0392)

-0.0333

(0.0333)

-0.0317

(0.0319)

-0.0921**

(0.0360)

-0.0984***

(0.0365)

-0.121***

(0.0353)

-0.122***

(0.0364)

Fixed Bonus

-0.0155

(0.0405)

-0.0166

(0.0414)

-0.0348

(0.0353)

-0.0420

(0.0354)

-0.0112

(0.0355)

-0.0128

(0.0353)

-0.0395

(0.0387)

-0.0390

(0.0384)

Wfa z-score*Performance Pay

-0.00492

(0.0212)

-0.00704

(0.0209)

-0.00589

(0.0169)

-0.00863

(0.0165)

-0.0281

(0.0202)

-0.0299

(0.0204)

-0.0227

(0.0227)

-0.0217

(0.0224)

Wfa z-score*Fixed Bonus

-0.0160

(0.0209) X

-0.0156

(0.0210)

-0.00959

(0.0181)

-0.00954

(0.0177)

0.0145

(0.0220) X

0.0157

(0.0215)

0.0238

(0.0217)

0.0211

(0.0215)

No controls

Child-level controls Mother-level controls Worker-level controls

X

X

X

X

X X

X

X

X

X

X X

N

6505

6505

4179

4179

6964

6964

4454

4454

Notes: Heteroscedasticity-consistent standard

errors accounting for clustering at the center level in parentheses. Data are from two

consecutive rounds of surveys carried out in October 2014 and January 2015 in columns (1) to (4) and from two consecutive rounds of

surveys carried out in January 2015 and April 2015 for columns (5) to (8). Attrited is a dummy variable that takes value equal to 1 if the child attrited from the sample between rounds (2) and (3) in columns (1) to (4) and between rounds (3) and (4) in columns (5) to (8). Performance Pay was promised to workers in November 2014 based on individual weight-for-age targets and was paid out in February 2015. Fixed Bonus was an ex-ante incentive of Rs. 200 per worker in November 2014. All dependent variables are the changes in a child's health indicator over the two consecutive rounds. Child-level controls include age and sex of child, mother controls include dummy variables for if mother identifies herself as scheduled caste, if mother identifies herself as Hindu, if there is a grandmother at home, if mother cannot read and write, if husband cannot read and write, if mother is a homemaker, if toilet is communal, if toilet has no flush, mother's age, total children in household, household income and an index of 13 fixed assets in the household. Worker-level controls are dummy variables for if worker identifies herself as scheduled caste, if worker identifies herself as Hindu, if worker is college-educated, worker's age and dummy variables for the availability of the following resources at the center: electricity, fan, helper, chart, blackboard, drinking water and toilet. *Significant at 10%, **Significant at 5%, ***Significant at 1%.

Table A3: Mean of health indicators across treatments and rounds

Block 1

Block 2

Control

Performance Pay

Fixed Bonus

Round 1

Weight

13.09

13.67

13.48

(1.95) (2.02) (1.98)

Round 2

Weight

13.47

13.74

13.73

(2.07) (2.09) (1.97)

Round 3

Weight

14.05

14.53

14.42

(2.05) (2.08) (1.99)

Round 4

Weight

13.90

14.49

14.29

(2.02) (2.07) (2.03)

Round 5

Weight

13.87

(2.09)

14.42

(2.26)

14.30

(2.04)

Round 1

Wfa Z-Score

-1.70

-1.51

-1.59

(0.82) (0.81) (0.83)

Round 2

Wfa Z-Score

-1.59

-1.47

-1.47

(0.85) (0.85) (0.83)

Round 3

Wfa Z-Score

-1.38

-1.18

-1.23

(0.83) (0.82) (0.82)

Round 4

Wfa Z-Score

-1.51

-1.23

-1.31

(0.81) (0.80) (0.88)

Round 5

Wfa Z-Score

-1.62

(0.79)

-1.32

(0.85)

-1.37

(0.83)

Round 1

Wfa Malnutrition

0.44

0.35

0.39

(0.50) (0.48) (0.49)

Round 2

Wfa Malnutrition

0.39

0.33

0.32

(0.49) (0.47) (0.47)

Round 3

Wfa Malnutrition

0.29

0.19

0.20

(0.45) (0.39) (0.40)

Round 4

Wfa Malnutrition

0.35

0.21

0.26

(0.48) (0.40) (0.44)

Round 5

Wfa Malnutrition

0.41

(0.49)

0.26

(0.44)

0.28

(0.45)

Table A4: Short term effects on health outcomes after introduction of treatments

(1)

Weight

(2)

Wfa z

(3)

Wfa mal

(4)

Weight

(5)

Wfa z

(6)

Wfa mal

*change in Dependent Variable*

Performance Pay

0.234***

(0.0618)

0.108***

(0.0302)

-0.0400*

(0.0222)

0.196***

(0.0696)

0.0899***

(0.0335)

-0.0451*

(0.0261)

Fixed Bonus

0.107

(0.0757) X

0.0490

(0.0352) X

-0.0185

(0.0221) X

0.103

(0.0860)

0.0474

(0.0405)

-0.0238

(0.0272)

No controls

Mother and child-level controls

X

X

X

N

5203

5169

5174

3528

3522

3524

Notes: Heteroscedasticity-consistent standard errors accounting for clustering at the center level in parentheses. Data are from two consecutive rounds

of surveys carried out in October 2014 and January 2015. Performance Pay was promised to workers in November 2014 based on individual weight-for- age targets and was paid out in February 2015. Fixed Bonus was an ex-ante incentive of Rs. 200 per worker in November 2014. All dependent variables are the changes in a child's health indicator over the two consecutive rounds. Weight is measured in kilograms. Wfa z is the weight-for-age z score given the child's sex and age. Wfh mal is an indicator for malnutrition as measured by weight-for-height z score and Wfa mal is an indicator based on weight-for-age z score. Mother and child-level controls include age and sex of child, a dummy variables for if mother identifies herself as scheduled caste, if mother identifies herself as Hindu, if there is a grandmother at home, if mother cannot read and write, if husband cannot read and write, if mother is a homemaker, if toilet is communal, if toilet has no flush, mother's age, total children in household, household income and an index of 13 fixed assets in the household. Worker-level controls are dummy variables for if worker identifies herself as scheduled caste, if worker identifies herself as Hindu, if worker is college-educated, worker's age and dummy variables for the availability of the following resources at the center: electricity, fan, helper, chart, blackboard, drinking water and toilet. *Significant at 10%, **Significant at 5%, ***Significant at 1%.

Table A5: Medium term effects on health outcomes

(1)

Weight

(2)

Wfa z

(3)

Wfa mal

(4)

Weight

(5)

Wfa z

(6)

Wfa mal

*change in Dependent Variable*

Performance Pay

0.157***

(0.0554)

0.0631**

(0.0266)

-0.0413**

(0.0165)

0.192***

(0.0707)

0.0793**

(0.0334)

-0.0487**

(0.0213)

Fixed Bonus

0.131**

(0.0657) X

0.0568*

(0.0321) X

-0.0262

(0.0189) X

0.159**

(0.0753)

0.0681*

(0.0371)

-0.0298

(0.0241)

No controls

Mother and child-level controls

X

X

X

N

3468

3436

3445

2303

2301

2302

Notes: Heteroscedasticity-consistent standard errors accounting for clustering at the center level in parentheses. Data are from two consecutive rounds

of surveys carried out in January 2015 and April 2015. Performance Pay was promised to workers in November 2014 based on individual weight-for-age targets and was paid out in February 2015. Another round of promises was made in February 2015 and payments were made in May 2015. Fixed Bonus was an ex-ante incentive of Rs. 200 in November 2014. All dependent variables are the changes in a child's health indicator over the two consecutive rounds. Weight is measured in kilograms. Wfa z is the weight-for-age z score given the child's sex and age. Wfh mal is an indicator for malnutrition as measured by weight-for-height z score and Wfa mal is an indicator based on weight-for-age z score. Mother and child-level controls include age and sex of child, a dummy variables for if mother identifies herself as scheduled caste, if mother identifies herself as Hindu, if there is a grandmother at home, if mother cannot read and write, if husband cannot read and write, if mother is a homemaker, if toilet is communal, if toilet has no flush, mother's age, total children in household, household income and an index of 13 fixed assets in the household. Worker-level controls are dummy variables for if worker identifies herself as scheduled caste, if worker identifies herself as Hindu, if worker is college-educated, worker's age and dummy variables for the availability of the following resources at the center: electricity, fan, helper, chart, blackboard, drinking water and toilet. *Significant at 10%,

**Significant at 5%, ***Significant at 1%.

Table A6: Fading-out effects on health outcomes after discontinuation of treatments

(1)

Weight

(2)

Wfa z

(3)

Wfa mal

(4)

Weight

(5)

Wfa z

(6)

Wfa mal

*change in Dependent Variable*

Performance Pay

0.101

(0.0772)

0.0327

(0.0354)

-0.0171

(0.0195)

0.0709

(0.0873)

0.0269

(0.0392)

-0.0218

(0.0238)

Fixed Bonus

0.0129

(0.0836) X

-0.00420

(0.0394) X

0.00587

(0.0192) X

0.0142

(0.0705)

0.00738

(0.0337)

0.00554

(0.0261)

No controls

Mother and child-level controls

X

X

X

N

3050

3022

3023

2230

2223

2224

Notes: Heteroscedasticity-consistent standard errors accounting for clustering at the center level in parentheses. Data are from two consecutive rounds

of surveys carried out in April 2015 and July 2015. Performance Pay was promised to workers in November 2014 based on individual weight-for-age targets and was paid out in February 2015. Another round of promises was made in February 2015 and payments were made in May 2015. Fixed Bonus was an ex-ante incentive of Rs. 200 per worker in November 2014. All dependent variables are the changes in a child's health indicator over the two consecutive rounds. Weight is measured in kilograms. Wfa z is the weight-for-age z score given the child's sex and age. Wfh mal is an indicator for malnutrition as measured by weight-for-height z score and Wfa mal is an indicator based on weight-for-age z score. Mother and child-level controls include age and sex of child, a dummy variables for if mother identifies herself as scheduled caste, if mother identifies herself as Hindu, if there is a grandmother at home, if mother cannot read and write, if husband cannot read and write, if mother is a homemaker, if toilet is communal, if toilet has no flush, mother's age, total children in household, household income and an index of 13 fixed assets in the household. Worker-level controls are dummy variables for if worker identifies herself as scheduled caste, if worker identifies herself as Hindu, if worker is college-educated, worker's age and dummy variables for the availability of the following resources at the center: electricity, fan, helper, chart, blackboard, drinking water and toilet.

*Significant at 10%, **Significant at 5%, ***Significant at 1%.

Table A7: Checking for effects on height

Between

R1 & R2

R2 & R3

R3 & R4

R4 & R5

R1 & R2

R2 & R3

R3 & R4

R4 & R5

(1)

Height

(2)

Height

(3)

Height

(4)

Height

(5)

Hfa z

(6)

Hfa z

(7)

Hfa z

(8)

Hfa z

*change in Dependent Variable*

Performance Pay

0.381

(0.480)

1.077**

(0.502)

-0.263

(0.375)

-0.0946

(0.382)

0.167*

(0.0930)

0.241**

(0.113)

-0.0675

(0.0838)

-0.0393

(0.0858)

Fixed Bonus

0.571

(0.494)

0.988*

(0.511)

-0.206

(0.332)

-0.546

(0.353)

0.166

(0.113)

0.223*

(0.115)

-0.0553

(0.0764)

-0.131

(0.0794)

No controls

Mother and child-level controls Worker-level controls

X

X

X

X

X

X

X

X

X

X

X

X

X

X

X

X

N

3721

3497

2286

2220

3707

3492

2285

2217

Notes: Heteroscedasticity-consistent standard errors accounting for clustering at the center level in parentheses. Data are from all five rounds of surveys carried out between July 2014 and July

2015. Performance Pay was promised to workers in November 2014 based on individual weight-for-age targets and was paid out in February 2015. Another round of promises was made in February 2015 and payments were made in May 2015. Fixed Bonus was an ex-ante incentive of Rs. 200 per worker in November 2014. All dependent variables are the changes in a child's height (in cms) over two consecutive rounds. Mother and child-level controls include age and sex of child, a dummy variables for if mother identifies herself as scheduled caste, if mother identifies herself as Hindu, if there is a grandmother at home, if mother cannot read and write, if husband cannot read and write, if mother is a homemaker, if toilet is communal, if toilet has no flush, mother's age, total children in household, household income and an index of 13 fixed assets in the household. Worker-level controls are dummy variables for if worker identifies herself as scheduled caste, if worker identifies herself as Hindu, if worker is college-educated, worker's age and dummy variables for the availability of the following resources at the center: electricity, fan, helper, chart, blackboard, drinking water and toilet. *Significant at 10%, **Significant at 5%, ***Significant at 1%.

Table A8: Checking for pre-trends in health outcomes between Baseline-I and Baseline-II

(1)

Weight

(2)

Wfa z

(3)

Wfh mal

(4)

Wfa mal

(5)

Weight

(6)

Wfa z

(7)

Wfh mal

(8)

Wfa mal

(9)

Weight

(10)

Wfa z

(11)

Wfh mal

(12)

Wfa mal

*change in Dependent Variable*

Performance Pay

-0.0428

(0.103)

0.000595

(0.0359)

0.0195

(0.0257)

-0.0307

(0.0219)

-0.0888

(0.124)

0.000222

(0.0405)

0.0185

(0.0292)

-0.0313

(0.0222)

-0.0991

(0.119)

-0.00620

(0.0411)

0.0199

(0.0280)

-0.0305

(0.0223)

0.135*

(0.0785) X

0.0736*

(0.0390) X

-0.00608

(0.0181) X

-0.0409

(0.0256) X

0.127

(0.0830)

0.0782*

(0.0402)

-0.0106

(0.0211)

-0.0404

(0.0277)

0.0971

(0.0884)

0.0694

(0.0423)

-0.00459

(0.0223)

-0.0305

(0.0285)

Fixed Bonus

No controls

Mother and child-level controls Worker-level controls

X

X

X

X

X

X

X

X

X

X

X

X

4674

4630

4393

4642

3744

3730

3533

3739

3744

3730

3533

3739

N

Notes: Heteroscedasticity-consistent standard errors accounting for clustering at the center level in parentheses. Data are from two consecutive rounds of Baseline

surveys carried out in July 2014 and October 2014. Performance Pay was promised to workers in November 2014 based on individual weight-for-age targets and was paid out in February 2015. Another round of promises was made in February 2015 and payments were made in May 2015. Fixed Bonus was an ex-ante incentive of Rs. 200 per worker in November 2014. All dependent variables are the changes in a child's health indicator over the two consecutive rounds. Weight is measured in kilograms. Wfa z is the weight-for-age z score given the child's sex and age. Wfh mal is an indicator for malnutrition as measured by weight-for-height z score and Wfa mal is an indicator based on weight-for-age z score. Mother and child-level controls include age and sex of child, a dummy variables for if mother identifies herself as scheduled caste, if mother identifies herself as Hindu, if there is a grandmother at home, if mother cannot read and write, if husband cannot read and write, if mother is a homemaker, if toilet is communal, if toilet has no flush, mother's age, total children in household, household income and an index of 13 fixed assets in the household. Worker-level controls are dummy variables for if worker identifies herself as scheduled caste, if worker identifies herself as Hindu, if worker is college- educated, worker's age and dummy variables for the availability of the following resources at the center: electricity, fan, helper, chart, blackboard, drinking water and toilet. *Significant at 10%, **Significant at 5%, ***Significant at 1%.

Table A9: Heterogeneity check for medium term effects

Total

children

<= 2

Total

children > 2

Fixed

assets > median

Fixed

assets < median

Literate

mother

Illiterate

mother

Literate

father

Illiterate

father

Boys

Girls

3-4 years

4-5 years

5-6 years

(1)

Weight

(2)

Weight

(3)

Weight

(4)

Weight

(5)

Weight

(6)

Weight

(7)

Weight

(8)

Weight

(9)

Weight

(10)

Weight

(11)

Weight

(12)

Weight

(13)

Weight

*change in Dependent Variable*

Performance Pay

0.154*

(0.0860)

0.161**

(0.0681)

0.140**

(0.0697)

0.134

(0.0970)

0.197**

(0.0920)

0.133*

(0.0785)

0.142

(0.102)

0.133*

(0.0754)

0.199*

(0.119)

0.120*

(0.0658)

0.200**

(0.0818)

0.217***

(0.0828)

0.107

(0.0878)

Fixed Bonus

0.133

(0.0851)

0.133

(0.0873)

0.186*

(0.0980)

0.125

(0.0884)

0.0145

(0.108)

0.141*

(0.0827)

0.149

(0.0943)

0.127

(0.0832)

0.234**

(0.111)

0.128*

(0.0740)

0.103

(0.0824)

0.227**

(0.0954)

0.101

(0.0774)

N

1681

1780

1202

1229

852

1766

940

2128

548

2070

1398

1346

1468

Notes: Heteroscedasticity-consistent standard errors accounting for clustering at the center level in parentheses. Data are from two consecutive rounds of surveys carried out in

January 2015 and April 2015. Performance Pay was promised to workers in November 2014 based on individual weight-for-age targets and was paid out in February 2015. Another round of promises was made in February 2015 and payments were made in May 2015. Fixed Bonus was an ex-ante incentive of Rs. 200 in November 2014. All dependent variables are the changes in a child's health indicator over the two consecutive rounds. Weight is measured in kilograms. Columns (1) and (2) split the sample by sex of child, columns (3)-(5) by age of child, column (6)-(9) by literacy of parents, columns (9) and (10) by total children in household less than or greater than the median of 2, and columns (12) and (13) by the median proportion of 13 fixed assets owned by household (46%). *Significant at 10%, **Significant at 5%, ***Significant at 1%.

Table A10: Mechanism of medium term effects - Quantity of interaction between worker and mother

(1)

(2)

(3)

Frequency of worker talking about child

(4)

(5)

(6)

Frequency of worker talking about child

(7)

(8)

(9)

Frequency of worker talking about child

Home visits

by worker

Center visits

by mother

Home visits

by worker

Center visits

by mother

Home visits

by worker

Center visits

by mother

*change in Dependent Variable*

Performance Pay

3.730***

(0.834)

-1.158

(1.144)

1.270

(1.353)

4.424***

(0.859)

-0.883

(1.190)

1.008

(1.394)

4.434***

(0.869)

-1.468

(1.229)

0.834

(1.323)

Fixed Bonus

4.106***

(0.892) X

-0.00817

(1.205) X

1.441

(1.402) X

4.495***

(0.918)

0.0936

(1.244)

1.250

(1.385)

4.570***

(0.929)

-0.841

(1.248)

1.876

(1.354)

No controls

Mother and child-level controls Worker-level controls

X

X

X

X

X

X

X

X

X

N

2758

2305

2607

2108

1753

1997

2108

1753

1997

Notes: Heteroscedasticity-consistent standard errors accounting for clustering at the center level in parentheses. Data are from two consecutive rounds of

surveys carried out in January 2015 and April 2015. Performance Pay was promised to workers in November 2014 based on individual weight-for-age targets and was paid out in February 2015. Another round of promises was made in February 2015 and bonuses were paid out in May 2015. Fixed Bonus was an ex-ante incentive of Rs. 200 per worker in November 2014. All dependent variables are the changes in the indicator over the two consecutive rounds. Home visits by worker are number of visits by Anganwadi worker to the household in the previous month as reported by the mother. Center visits by mother are mother's visits to the Anganwadi in the previous month as reported by the mother. Frequency of worker talking about child is the number of times worker spoke about child with mother in the previous month as reported by the mother. Mother and child-level controls include age and sex of child, a dummy variables for if mother identifies herself as scheduled caste, if mother identifies herself as Hindu, if there is a grandmother at home, if mother cannot read and write, if husband cannot read and write, if mother is a homemaker, if toilet is communal, if toilet has no flush, mother's age, total children in household, household income and an index of 13 fixed assets in the household. Worker-level controls are dummy variables for if worker identifies herself as scheduled caste, if worker identifies herself as Hindu, if worker is college-educated, worker's age and dummy variables for the availability of the following resources at the center: electricity, fan, helper, chart, blackboard, drinking water and toilet. *Significant at 10%, **Significant at 5%, ***Significant at 1%.

Table A11: Mechanism of medium term effects - Quality of information provided by worker to mother

(1)

Nutrition

(2)

Hygiene

(3)

Chart

(4)

Scare

(5)

Nutrition

(6)

Hygiene

(7)

Chart

(8)

Scare

(9)

Nutrition

(10)

Hygiene

(11)

Chart

(12)

Scare

*change in Dependent Variable*

Performance Pay

-0.231***

(0.0824)

0.0667

(0.0875)

-0.355***

(0.108)

-0.119

(0.0808)

-0.275***

(0.0763)

0.108

(0.0871)

-0.378***

(0.103)

-0.139*

(0.0826)

-0.306***

(0.0802)

0.0843

(0.0805)

-0.341***

(0.105)

-0.133

(0.0876)

Fixed Bonus

-0.120

(0.0760) X

0.164**

(0.0759) X

-0.433***

(0.0985) X

0.0672

(0.0773) X

-0.174**

(0.0784)

0.222***

(0.0785)

-0.496***

(0.0987)

-0.000201

(0.0820)

-0.177**

(0.0747)

0.246***

(0.0795)

-0.487***

(0.0981)

-0.0122

(0.0916)

No controls

Mother and child-level controls Worker-level controls

X

X

X

X

X

X

X

X

X

X

X

X

N

2645

2645

2645

2645

2024

2024

2024

2024

2024

2024

2024

2024

Notes: Heteroscedasticity-consistent standard errors accounting for clustering at the center level in parentheses. Data are from two consecutive rounds of surveys

carried out in January 2015 and April 2015. Performance Pay was promised to workers in November 2014 based on individual weight-for-age targets and was paid out in February 2015. Another round of promises was made in February 2015 and bonuses were paid out in May 2015. Fixed Bonus was an ex-ante incentive of Rs. 200 per worker in November 2014. All dependent variables are the changes in the indicator over the two consecutive rounds. All dependent variables are the changes in an indicator over the two consecutive rounds. Nutrition is a dummy variable equal to 1 if, in the last month, the worker spoke to the mother about her child's nutrition. Hygiene is a dummy variable equal to 1 if, in the last month, the worker talked with the mother about maintaining child's hygiene. Chart is a dummy variable equal to 1 if, in the last month, the worker showed the mother a growth chart. Scare is a dummy variable equal to one if, in the last month, the worker scared the mother with consequences of malnutrition. Mother and child-level controls include age and sex of child, a dummy variables for if mother identifies herself as scheduled caste, if mother identifies herself as Hindu, if there is a grandmother at home, if mother cannot read and write, if husband cannot read and write, if mother is a homemaker, if toilet is communal, if toilet has no flush, mother's age, total children in household, household income and an index of 13 fixed assets in the household. Worker-level controls are dummy variables for if worker identifies herself as scheduled caste, if worker identifies herself as Hindu, if worker is college-educated, worker's age and dummy variables for the availability of the following resources at the center: electricity, fan, helper, chart, blackboard, drinking water and toilet. *Significant at 10%,

**Significant at 5%, ***Significant at 1%.

Table A12: Mechanisms of medium term effects - Diet at home

(1)

Milk

(2)

Green veg

(3)

Dessert

(4)

Porridge

(5)

Milk

(6)

Green veg

(7)

Dessert

(8)

Porridge

(9)

Milk

(10)

Green veg

(11)

Dessert

(12)

Porridge

*change in Dependent Variable*

Performance Pay

0.110***

(0.0274)

-0.00291

(0.0303)

-0.0267

(0.0596)

0.233***

(0.0654)

0.125***

(0.0291)

-0.00165

(0.0362)

-0.0963

(0.0612)

0.185***

(0.0686)

0.125***

(0.0317)

-0.0212

(0.0430)

-0.116*

(0.0618)

0.169**

(0.0657)

Fixed Bonus

0.0806***

(0.0233)

-0.0286

(0.0345)

-0.0397

(0.0632)

-0.00832

(0.0607)

0.0889***

(0.0274)

-0.0323

(0.0397)

-0.0855

(0.0661)

-0.0377

(0.0668)

0.0902***

(0.0284)

-0.0462

(0.0452)

-0.0867

(0.0673)

-0.0338

(0.0708)

No controls

Mother and child-level controls Worker-level controls

X

X

X

X

X

X

X

X

X

X

X

X

X

X

X

X

N

2007

2011

1994

1992

1517

1518

1505

1508

1517

1518

1505

1508

Notes: Heteroscedasticity-consistent standard errors accounting for clustering at the center level in parentheses. Data are from two consecutive rounds of surveys

carried out in January 2015 and April 2015. Performance Pay was promised to workers in November 2014 based on individual weight-for-age targets and was paid out in February 2015. Another round of promises was made in February 2015 and bonuses were paid out in May 2015. Fixed Bonus was an ex-ante incentive of Rs. 200 per worker in November 2014. All dependent variables are the changes in an indicator over the two consecutive rounds. Milk, Green veg, Dessert (traditional) and Porridge are dummy variables equal to 1 if the mother reports feeding these items at least twice a week to her child. Mother and child-level controls include age and sex of child, a dummy variables for if mother identifies herself as scheduled caste, if mother identifies herself as Hindu, if there is a grandmother at home, if mother cannot read and write, if husband cannot read and write, if mother is a homemaker, if toilet is communal, if toilet has no flush, mother's age, total children in household, household income and an index of 13 fixed assets in the household. Worker-level controls are dummy variables for if worker identifies herself as scheduled caste, if worker identifies herself as Hindu, if worker is college-educated, worker's age and dummy variables for the availability of the following resources at the center: electricity, fan, helper, chart, blackboard, drinking water and toilet. *Significant at 10%, **Significant at 5%, ***Significant at 1%.

Table A13: Checking pre-trends for mechanisms

Notes: Heteroscedasticity-consistent standard errors accounting for clustering at the center level in parentheses. Data are from two consecutive rounds of surveys

carried out in July 2014 and October 2014. Performance Pay was promised to workers in November 2014 based on individual weight-for-age targets and was paid out in February 2015. Another round of promises was made in February 2015 and bonuses were paid out in May 2015. Fixed Bonus was an ex-ante incentive of Rs. 200 per worker in November 2014. All dependent variables are the changes in the indicator over the two consecutive rounds. Home visits by worker are number of visits by Anganwadi worker to the household in the previous month as reported by the mother. Center visits by mother are mother's visits to the Anganwadi in the previous month as reported by the mother. Frequency of worker talking about child is the number of times worker spoke about child with mother in the previous month as reported by the mother. Nutrition is a dummy variable equal to 1 if, in the last month, the worker spoke to the mother about her child's nutrition. Hygiene is a dummy variable equal to 1 if, in the last month, the worker talked with the mother about maintaining child's hygiene. Chart is a dummy variable equal to 1 if, in the last month, the worker showed the mother a growth chart. Scare is a dummy variable equalto one if, in the last month, the worker scared the mother with consequences of malnutrition. Milk, Green veg, Dessert (traditional) and Porridge are dummy variables equal to 1 if the mother reports feeding these items at least twice a week to her child. Mother and child-level controls include age and sex of child, a dummy variables for if mother identifies herself as scheduled caste, if mother identifies herself as Hindu, if there is a grandmother at home, if mother cannot read and write, if husband cannot read and write, if mother is a homemaker, if toilet is communal, if toilet has no flush, mother's age, total children in household, household income and an index of 13 fixed assets in the household. Worker- level controls are dummy variables for if worker identifies herself as scheduled caste, if worker identifies herself as Hindu, if worker is college-educated, worker's age and dummy variables for the availability of the following resources at the center: electricity, fan, helper, chart, blackboard, drinking water and toilet. *Significant at 10%, **Significant at 5%, ***Significant at 1%.

(1) (2) (3)

Frequency

Center of worker Home visits visits by talking

*change in Dependent Variable* by worker mother about child

(4) (5) (6) (7)

Nutrition Hygiene Chart Scare

(8) (9) (10) (11)

Milk Green veg Dessert Porridge

Performance Pay 0.120 0.633 -1.419

(1.144) (1.070) (0.880)

Fixed Bonus 1.056 -1.020 -1.571

(1.105) (1.322) (0.982)

Mother and child-level controls X X X Worker-level controls X X X

N 3412 3002 3178

0.0294 0.104 0.104 0.0552

(0.0756) (0.0887) (0.0823) (0.0664)

0.0115 0.0662 0.230*** 0.0703

(0.0727) (0.0978) (0.0774) (0.0589) X X X X

X X X X

3329 3329 3329 3329

0.00195 -0.0700* 0.0600 -0.0229

(0.0168) (0.0421) (0.0868) (0.0410)

-0.0191 -0.0394 0.0278 -0.0167

(0.0160) (0.0480) (0.0675) (0.0559) X X X X

X X X X

2280 2276 2241 2235

Table A14: Lee (2009) treatment effect bounds

Weight change between Rounds 2 and 3

Performance Pay

lower upper

Coef.

0.057

0.388

Std. Err.

0.052

0.051

z

1.100

7.630

P>|z|

0.270

0.000

Fixed Bonus

lower upper

Coef.

0.044

0.181

Std. Err.

0.072

0.084

z

0.610

2.140

P>|z|

0.540

0.032

Weight change between Rounds 3 and 4

Performance Pay

lower upper

Coef.

0.050

0.275

Std. Err.

0.049

0.047

z

1.030

5.830

P>|z|

0.305

0.000

Fixed Bonus

lower upper

Coef.

0.115

0.147

Std. Err.

0.110

0.117

z

1.040

1.260

P>|z|

0.298

0.207

Table A15:

Net gains and transitions between malnutrition categories by treatment arm (percent of children)

Short-term (from R2 to R3)

*N*

*(# of obs.)*

Net Gain

Improved

No Change

Worsened

Performance Pay

Fixed Bonus Control

13.3

11.1

9.3

16.4

15.5

15.0

80.6

80.2

79.3

3.1

4.4

5.7

*850*

*826*

*3498*

Medium-term (from R3 to R4)

*N*

*(# of obs.)*

Net Gain

Improved

No Change

Worsened

Performance Pay

Fixed Bonus Control

-3.0

-4.6

-7.2

3.2

5.8

5.9

90.6

83.9

81.1

6.2

10.3

13.0

*855*

*834*

*1756*

Table A16: Regressions clustering Moulton standard errors to correct for small sample size

Short term

Medium term

(1)

Weight

(2)

Wfa z

(3)

Wfa Mal

(4)

Weight

(5)

Wfa z

(6)

Wfa Mal

*change in Dependent Variable*

Performance Pay

0.219**

(0.0977)

0.101**

(0.0460)

-0.0561**

(0.0274)

0.231***

(0.0764)

0.0976***

(0.0371)

-0.0522**

(0.0236)

Fixed Bonus

0.123

(0.0968) X

X 3528

0.0557

(0.0455) X

X 3522

-0.0333

(0.0271) X

X 3524

0.196**

(0.0765) X

X 2303

0.0878**

(0.0372) X

X 2301

-0.0341

(0.0237) X

X 2302

Mother and child-level controls

Worker-level controls N

Notes: Heteroscedasticity-consistent

Moulton standard

errors accounting

for clustering

at the center

level in parentheses.

Data are from two

consecutive rounds of surveys carried out in October 2014 and January 2015. Performance Pay was promised to workers in November 2014 based on

individual weight-for-age targets and was paid out in February 2015. Fixed Bonus was an ex-ante incentive of Rs. 200 per worker in November 2014. All dependent variables are the changes in a child's health indicator over the two consecutive rounds. Weight is measured in kilograms. Wfa z is the weight- for-age z score given the child's sex and age. Wfa mal is an indicator based on weight-for-age z score. Mother and child-level controls include age and sex of child, a dummy variables for if mother identifies herself as scheduled caste, if mother identifies herself as Hindu, if there is a grandmother at home, if mother cannot read and write, if husband cannot read and write, if mother is a homemaker, if toilet is communal, if toilet has no flush, mother's age, total children in household, household income and an index of 13 fixed assets in the household. Worker-level controls are dummy variables for if worker identifies herself as scheduled caste, if worker identifies herself as Hindu, if worker is college-educated, worker's age and dummy variables for the availability of the following resources at the center: electricity, fan, helper, chart, blackboard, drinking water and toilet. *Significant at 10%, **Significant at 5%, ***Significant at 1%.

Table A17: Attendance of worker as measured through announced visit by independent supervisor between rounds

(1)

(2)

(3)

(4)

(5)

(6)

(7)

(8)

2014

2015

August September November December

February March May June

Performance Pay

0.0336

(0.0774)

-0.0303

(0.0862)

0.274***

(0.0519)

0.132

(0.0855)

-0.0809

(0.0947)

-0.0702

(0.103)

0.102

(0.0850)

0.175

(0.145)

Fixed Bonus

0.0733

(0.0702)

0.130*

(0.0693)

0.186***

(0.0703)

0.133

(0.0869)

0.130*

(0.0771)

0.194**

(0.0773)

0.0596

(0.0865)

0.0791

(0.144)

Control

0.795***

(0.0319)

0.730***

(0.0347)

0.662***

(0.0371)

0.576***

(0.0389)

0.721***

(0.0502)

0.685***

(0.0513)

0.765***

(0.0464)

0.353***

(0.0532)

N

11689

12197

12325

12249

8035

7342

7176

5514

Notes: Heteroscedasticity-consistent standard errors accounting for clustering at the center level in parentheses. Data are from two consecutive rounds

of surveys carried out in October 2014 and January 2015. Performance Pay was promised to workers in November 2014 based on individual weight-for- age targets and was paid out in February 2015. Another round of promises was made in February 2015 and bonuses were paid out in May 2015. Fixed Bonus was an ex-ante incentive of Rs. 200 per worker in November 2014. All dependent variables are the dummy variables for worker attendance during an unannounced visit in the specified inter-survey period. No other controls are included. *Significant at 10%, **Significant at 5%, ***Significant at 1%.

**APPENDIX - FIGURES**

Figure A1: Change in weight between rounds 2 and 3 (kgs)

Figure A2: Change in weight between rounds 3 and 4 (kgs)

Figure A3: Effects of performance pay on weight between R2 and R3

Figure A4: Effects of performance pay on weight between rounds 3 and 4

Figure A5: Worker Attendance in the Control group

Figure A6 Worker Attendance in the Performance Pay treatment

Figure A7: Worker Attendance in the Fixed Bonus treatment

Figure A8: Expected Performance Pay (as a share of maximum incentive)

Figure A9: Expected incentive (Rupees)
